# POLICY INSIGHTS FROM THE EMF 32 STUDY ON U.S. CARBON TAX SCENARIOS*

**DOI:** 10.1142/S2010007818400031

**Published:** 2018-03-20

**Authors:** ALEXANDER R. BARRON, ALLEN A. FAWCETT, MARC A. C. HAFSTEAD, JAMES R. MCFARLAND, ADELE C. MORRIS

**Affiliations:** † Environmental Science and Policy Program, Smith College 44 College Lane, Northampton, MA 01063, USA; ‡ U.S. Environmental Protection Agency, 1200 Pennsylvania Avenue NW Washington, DC 20460, USA; § Resources for the Future, 1616 P St. NW, Washington, DC 20036, USA; ¶ Brookings Institution, 1775 Massachusetts Ave, NW Washington, DC 20036, USA

**Keywords:** Climate change, model comparison, decarbonization, CGE models

## Abstract

The Stanford Energy Modeling Forum exercise 32 (EMF 32) used 11 different models to assess emissions, energy, and economic outcomes from a plausible range of economy-wide carbon price policies to reduce carbon dioxide (CO_2_) emissions in the United States. Here we discuss the most policy-relevant results of the study, mindful of the strengths and weaknesses of current models. Across all models, carbon prices lead to significant reductions in CO_2_ emissions and conventional pollutants, with the vast majority of the reductions occurring in the electricity sector. Importantly, emissions reductions do not significantly depend on the rebate or tax cut used to return revenues to the economy. Expected economic costs, as modeled by either GDP or welfare, are modest, but vary across models. These costs are offset by benefits from avoided climate damages and health benefits from reductions in conventional air pollution. Using revenues to reduce preexisting capital or labor taxes reduces costs in most models relative to lump-sum rebates, but the size of the cost reductions varies significantly. Devoting at least some revenue to household rebates can significantly reduce adverse impacts on low income households. Carbon prices at $25/ton or even lower levels cause significant shifts away from coal as an energy source with responses of other energy sources highly dependent upon technology cost assumptions. Beyond 2030, we conclude that model uncertainties are too large to make quantitative results useful for near-term policy design. We close by describing recommendations for policymakers on interacting with model results in the future.

## 1. Introduction

Policymakers seeking to reduce greenhouse gas (GHG) emissions have at their disposal a wide range of possible policy approaches and tools, including voluntary programs, prizes, mandates, command and control regulations, subsidies, and market-based policies (taxes and emissions trading programs) (Fischer and Newell, 2008; Goulder and Parry, 2008). Of these, economists widely regard market-based policies which impose a price on emissions as the most cost-effective and efficient approach. The Intergovernmental Panel on Climate Change, the World Bank, OECD, and the International Monetary Fund have all endorsed carbon pricing as a cost-effective tool for reducing emissions (IPCC, 2014; Davenport, 2016). Others have noted that supporters of carbon taxes might not even need to be particularly concerned about climate change; they could simply be interested in reducing other tax rates and/or regulations (Stelzer, 2014). Support for carbon pricing policies has been growing. Some form of carbon price is in place or under development in 40 national and 25 subnational jurisdictions (World Bank, Ecofys and Vivid Economics, 2017). The Carbon Pricing Leadership Coalition connects a wide range of countries (e.g., Germany and Ivory Coast) and businesses (e.g., Coca Cola and Shell) working on carbon pricing approaches (Carbon Pricing Leadership Coalition, 2017). The Climate Leadership Council, consisting of a wide range of businesses and led by a number of prominent Republicans, recently released a proposal for an economy-wide carbon tax in the United States (Baker *et al*., 2017).

Once policymakers settle on a given policy tool, such as a carbon tax, a host of questions arise about the appropriate level of the tax, the emissions reductions likely to be achieved, the use of the revenue, the macroeconomic impacts, distributional impacts, and whether supplemental policies are needed (Morris, 2016). Given the complexity of the energy system and its links to the economy, modeling is one of the best available tools to understand possible outcomes of a policy and explore trade-offs between design choices. However, all models come with strengths and limitations. As the statistician George Box once put it: “All models are wrong, but some are useful” (Box and Draper, 1987). One way to obtain a more robust understanding of the likely impacts of a policy is to analyze it with several different models and to identify results that are consistent across a range of model types and designs. The Stanford Energy Modeling Forum (EMF) has been conducting model inter-comparison exercises on policy-relevant topics since the late 1970s. More detail on the history of the Energy Modeling Forum and past modeling exercises can be found in Fawcett *et al*. (2014).

The purpose of this particular Energy Modeling Forum exercise (EMF 32) is to employ multiple models to compare outcomes of a range of plausible carbon price policies in the United States. (We will interchangeably use the term “carbon tax” and the more general term “carbon price” as other carbon pricing policies can produce similar outcomes). A community of 17 modeling teams gathered to run analyses of U.S. climate policies; some policies applied only to the U.S. electricity sector and some applied broadly to all fossil fuel-related CO_2_ emissions throughout the U.S. economy. Modeling teams coordinated assumptions about the future without new climate policies (the reference case) and applied the policies in roughly consistent ways. Some modelers analyzed all the scenarios; others only a few. Bistline *et al*. (2018) summarize the policy-relevant insights from the work of teams that analyzed policies confined to the power sector. This special issue of Climate Change Economics presents the findings of the 11 teams that ran economy-wide U.S. carbon tax scenarios.

This paper is a nontechnical summary that focuses on the implications for policy design. Also in this special issue of *Climate Change Economics*, 10 individual modeling teams present insights from their specific models, as summarized by Fawcett *et al*. (this issue). McFarland *et al*. (this issue) provide a technical overview of the study design and core results. The reader should turn to that paper for a discussion of model-specific results and how and why they differ. Two other papers focus on particular crosscutting issues. Caron *et al*. (this issue) focus on the distributional effects of a carbon price across income classes and regions within the United States, and Macaluso *et al*. (this issue) detail results in specific sectors of the economy with a focus on energy production and international spillovers of unilateral U.S. policy. Although this paper is a stand-alone summary, we direct the reader to more detailed papers in this issue. We also draw on insights from past EMF exercises and the authors’ collective experiences engaging with the policymaking process.

This study examines potential implications of an economy-wide carbon pricing policy by varying two key parameters: the trajectory of the carbon price and the use of the revenue. All of the scenarios apply the carbon tax to all fossil fuel CO_2_ emissions, which represent roughly 77% of overall gross U.S. GHG emissions (EPA, 2017a). While we call it an “economy-wide” carbon tax, it omits carbon emissions from certain industrial processes (e.g., cement), terrestrial ecosystems, and emissions of other GHGs (e.g., methane emissions).

Two approaches determine the carbon price trajectory. The first approach sets a fixed tax trajectory in which the tax begins in 2020 at either $25 or $50 and rises at either 1% or 5% per year, leveling off in 2050.^1^Most of this paper focusses on these four “core” price trajectories. In other scenarios, the modelers sought to find the price trajectory that would achieve a given emissions target in a particular year. For example, one scenario solves for the carbon tax path that achieves a 26% reduction in economy-wide fossil CO_2_ emissions in 2025 relative to 2005 levels, a target consistent with the U.S. Nationally Determined Contribution submitted under the Paris Accord (UNFCCC, 2016) and a related scenario extends that to also achieve an 80% reduction by 2050, consistent with the G8 Major Economies Forum commitments in 2009 (Wintour and Elliott, 2009). Yet another scenario solves for the carbon tax imposed solely on fossil carbon used in electricity production that achieves the electricity sector emissions level in 2030 that EPA projected under the agency’s Clean Power Plan as finalized in 2015.^2^

Some models also examined a price trajectory based on the social cost of carbon (SCC) used for analyzing federal regulations in 2015 and 2016 (at a 3% discount rate). We do not report that scenario here because it is very similar to the price trajectory starting at $50 and rising at 1%. In addition, that version of the SCC is no longer in use by the federal government (see Sec. 5), and the National Academies of Sciences recently issued a report suggesting multiple short- and long-term improvements to the SCC estimate, which would likely change the value in the future (National Academies of Sciences, Engineering, and Medicine, 2017).

Policymakers can use the revenue generated by the carbon tax in any number of ways (e.g., cutting other taxes, new spending on research and development, reducing the deficit). This study focusses on “revenue neutral” approaches, in which we hold government spending to baseline levels and return net revenue to households either as direct rebates (also called lump-sum transfers) or via cuts in the marginal tax rates on capital or labor income. Some scenarios combine rebates to households and tax rate cuts. For example, one scenario gives rebates to the 20% of households with the lowest incomes such that they are not made worse off and then splits the remainder of revenue between labor and capital tax cuts. Another provides rebates to the 20% of households with the lowest incomes sufficient to hold their welfare to baseline levels and then tried to leave every other income bracket better off. Further details on those policy scenarios appear in Caron *et al*. (this issue).

Modeling studies like this one generally focus on how the policy scenarios differ from each other and from an otherwise equivalent future that does not include the policy, i.e., a reference scenario. To the extent feasible, modelers in this project calibrated their model’s reference scenarios to the U.S. Energy Information Administration’s (EIA) Annual Energy Outlook (AEO) 2016 Early Release Case (EIA, 2016a). That AEO reference scenario takes into account existing, implemented federal and state policies such as the EPA/DOT standards for light-duty vehicles, California’s AB 32 climate law, and state renewable portfolio standards, but it does not include the Clean Power Plan. Modelers were not asked to consider alternative reference scenarios (such as with alternative economic growth rates, energy efficiency improvements, renewable energy costs, natural gas prices, etc.); alternative reference scenarios may increase or decrease the expected emissions reductions, welfare, or other outcomes of the policy scenarios.^3^

This paper has 10 sections. Section 2 explains the value of modeling climate policies and reviews important caveats in interpreting results. Section 3 discusses the carbon price scenarios and their associated near-term emissions reductions (through 2030). Section 4 discusses the challenges involved in longer-term (post-2030) analyses. Section 5 discusses the economic outcomes of the policy scenarios, including the likely impacts on revenue and welfare. Section 6 discusses how results vary across households at different levels of income and in different regions of the country. Section 7 examines emissions and output prices in key sub-sectors of the economy (electricity generation, residential households, industry, etc.). Section 8 briefly reviews findings on trade and competitiveness. Section 9 discusses additional caveats and limitations of this work. Section 10 concludes by offering overarching recommendations to policymakers.

## 2. General Considerations on Modeling Climate Policy

Any policy that significantly reduces U.S. GHG emissions will require important shifts in the energy system that powers the American economy, especially the 81% of energy that currently comes from fossil fuels (EIA, 2016b). Given the complexity of these energy systems and their role in the economy, modeling is key to investigating the advantages and disadvantages of different policy options. Just within the electricity sector, specialized models are necessary to account for a policy’s effect on the multiple fuel types, generation technologies, and grid networks that power different regions of the country. Models are also useful tools to anticipate the interactions between these many pieces. For example, a price on carbon changes the relative prices of different fuels in relation to their carbon intensity, which in turn changes the mix of energy sources in production, and the prices of goods and services with different amounts of carbon in their supply chain. It can also change wages, returns to capital investments, and growth in different sectors, and some of the impacts can spill over beyond U.S. borders through trade, currency, and investment flows. Anticipating this web of interactions within and between sectors is important to understanding the full set of potential outcomes of a policy. At the same time, the complexities across space, time, and sectors, as well as the obvious uncertainties in predicting policy, market, and technological conditions make accurately capturing these dynamics challenging.

To capture the range of potential outcomes that might occur under a carbon tax or any other major climate policy, it is useful to deploy a variety of models that represent different degrees of detail in different dimensions of energy systems and the overall economy. For example, some models divide the United States into over 350 regions, whereas others represent the United States as a single region in a multi-region global economy. Some include only CO_2_ while others represent additional GHGs and/or conventional air pollutants. Models may represent individual economic sectors in detail or divide households by income class, whereas others represent sectors and households as a whole. Greater detail has its tradeoffs. For example, analyzing the integration of renewable generation into the electricity grid can require representing supply and demand on a fine temporal scale. At the same time, more detail can make models slow to run, hard to calibrate with accurate data, hard to maintain, more subject to uncertainty from parameter estimates, and harder to interpret. That means different models tend to have different areas of relative strength, making multi-model analyses particularly useful for policy decisions with potentially complex economic impacts. Consistent with this focus, figures throughout the text are designed to highlight the patterns across the full range of models and are therefore generally not labeled with model names to avoid undue complexity. Details on model-specific results can be found in McFarland *et al*. (this issue) and in the other papers in this issue, as enumerated in Fawcett *et al*. (this issue).

As noted in Sec. 1, a particular focus of the economy-wide carbon tax scenarios in EMF 32 is the set of economic outcomes associated with different uses of the revenue. For this purpose, computational general equilibrium (CGE) models are especially useful. CGE models generally incorporate a large collection of equations that represent the different parts of the overall economy, such as production and consumption of different inputs (such as capital, labor, energy, and materials) in different sectors (such as different industries and services). These models use economic data to set up these equations, so that they can estimate how, for example, households will change buying patterns or companies will change production processes when energy prices change, along with the ripple effects through the rest of the economy. Importantly, they can also model how firms and households may respond to changes in different tax rates (e.g., by investing or working more (or less)). In contrast to CGE models, “partial equilibrium” models use a range of approaches to approximate the same dynamic, but tend not to capture the full range of a policy’s macroeconomic spillovers. Details of the models used in the EMF 32 carbon tax study appear in McFarland *et al*. (this issue). A breakdown of some of the major features of these types of models is reviewed elsewhere (Hedenus *et al*., 2013; SAB, 2017).

Modeling necessarily involves making assumptions about how the world works, and each assumption has its own implications. Some of the results can be robust across different assumptions and modeling platforms, and others can be quite sensitive. Here we present some examples of caveats that are particularly relevant to this study.

### Environmental benefits are not included

The primary policy goal of a price on carbon is to lower emissions that can drastically change the earth’s climate and acidify the ocean. A complete measure of welfare outcomes of a climate policy would include both its costs and the benefits from avoiding climate damages as well as lower (conventional) air pollution. Although we discuss these benefits at a high level below, the models do not fully account for their ripple effects through the economy, compounding over time, the way they do other outcomes (i.e., cost). Approaches for calculating these benefits in economic terms are still being developed (SAB, 2017).

### Constancy and predictability

For simplicity and ease of interpretation, we model policies that endure indefinitely, and we hold all other policies in the economy to their existing trajectory. In reality, policymakers can abruptly rescind or tighten policies, and both the domestic and global policy landscapes are constantly changing. Moreover, states can take action that interacts with the outcomes of federal policies. In the real world, economic actors will take all of these potential policy changes into account and dynamically adjust their behavior in ways that predictions based on historical patterns cannot anticipate (Lucas, 1976).

### Unrealistic foresight

CGE models typically represent at least some economic actors as having the ability to anticipate coming conditions and optimize through the end of the modeling horizon (past 2050).^4^ This better represents savings and investment behavior, but the assumption gives economic agents in the model unrealistic knowledge and certainty about future policies. For example, a business may reduce emissions more in 2020 than the carbon tax rate that year might warrant in anticipation of higher carbon prices decades away. Real businesses and households may have shorter planning horizons, for example, because they have limited access to financing or they imperfectly predict future conditions (although they can and do anticipate future possible changes).

### A highly stylized economy

Economic models generally depict the economy as stylized and efficient. Although models represent some existing distortions in the economy (e.g., capital taxes that reduce economic growth) and can thus illustrate the benefits of reducing those distortions, most models do not include a vast range of inefficiencies (financing constraints, monopolistic competition, undersupplied R&D, behavioral barriers to energy efficiency investments, etc.) and therefore cannot capture all of the interactions of a carbon tax with these various inefficiencies. For example, a carbon tax could induce investments in basic research and development that is currently undersupplied, but none of the models used here would fully capture that result.

### Full employment

The sets of CGE models included in this exercise are “full employment” models. In full employment models, wages adjust such that total labor supply equals total labor demand (at different speeds depending upon the model). A carbon tax may change the total labor supplied and the allocation of that labor across different sectors, but the shifts cannot be interpreted as unemployment (i.e., a disconnect between the supply and demand for labor). An often repeated interpretation of full employment models is that “everyone who wants a job has one.” That means that the models are generally not useful in understanding how many workers will be dislocated or gain employment in different regions or industries. A recent review by EPA’s independent Science Advisory Board found: “[A] full-employment model can’t look at impacts that are directly related to full employment. Some implications of that limitation are obvious: a full-employment model cannot examine effects on unemployment” (SAB, 2017). Models are being developed to include unemployment impacts, but these models are in their infancy (Hafstead and Williams, 2016).

### Scenarios, not forecasts

Ideally, policymakers would like to know the exact (or most likely) outcomes of a policy in the future, such as the price of electricity in a certain time and place, i.e., a forecast. However, our purpose here is not forecasting, but rather to investigate how alternative, arguably plausible, versions of the future compare with each other so that we can distill important insights for policymakers. Economic projections involve considerable uncertainties owing to a variety of factors, including long-time horizons, human behavior, technology, evolving policies, and all sorts of hard-to-predict conditions such as interest rates, economic growth, factor prices, and population growth (Weyant, 2017). Few climate policies have been in place long enough to assess the accuracy of modeled projections after the fact (but see Ellerman *et al*., 2010; Murray and Rivers, 2015; Olson *et al*., 2016), but even business-as-usual projections have often differed significantly from the actual trends in energy demand and renewable energy deployment (Craig *et al*., 2002; EIA, 2015; Gilbert and Sovacool, 2016). As the U.S. Energy Information Administration states “Projections...are not predictions of what will happen, but rather modeled projections of what may happen given certain assumptions and methodologies” (EIA, 2017). Thus, here we emphasize broad insights about policy design while noting the potential for a range of possible outcomes.

### Data/baseline calibration

Modelers calibrate to recently available data. A model calibrated to 2005, prior to the shale boom, would generate different results than an otherwise identical model calibrated to 2015. As an example, EIA’s AEO2016 projections of the reference case for the commercial, residential, and industrial sectors were calibrated to data from 2003, 2009, and 2010, respectively (EIA, 2016a) (AEO2017 uses 2012 commercial data). As models in the EMF exercise were asked to calibrate their models to the AEO2016 reference case, this generally means that the models do better at capturing small shifts in the near future than big shifts in the distant future.

These calibrations sometimes miss new technologies or shifts in behavior that have already occurred due to lags in data availability (e.g., the full shale gas boom, advances in heat pumps or building controls, and cheaper solar options). By definition, these calibrations will not be able to anticipate future shifts in technology or behavior that may be important to the economic response to carbon taxation (although some modelers may attempt to add more speculative responses for emerging technologies (e.g., CCS)). As we will discuss in Sec. 4, these calibrations can affect the degree to which the models can capture reductions in other sectors.

In addition, the baseline does not reflect the recent tax legislation in the United States, which substantially changes the statutory corporate tax rate and other revenue variables. This changes both baseline tax parameters and baseline debt projections, which in turn could affect baseline economic growth and the efficiency gains from the tax swap scenarios, among other things.

### Induced innovation

A carbon tax may spur innovation by increasing incentives for research and development on low-carbon or energy-efficient technologies relative to the reference case. Indeed, that may be one of its most important outcomes. CGE models represent technological change at the sector level through empirically-based estimates of production processes and at the technology level by introducing new technologies (e.g., CCS technologies). However, these models generally do not represent induced research and development spending and the associated spillovers. That means the results here may understate the environmental effectiveness of the policies; the problem is that we do not know by how much.^5^

Recognizing all of the limitations defined above, we endeavor to balance those limitations with the need to make policy decisions despite uncertainty.

## 3. Emissions Outcomes Over the Near Term

### 3.1. Carbon dioxide emissions reductions

**Figure 1.**
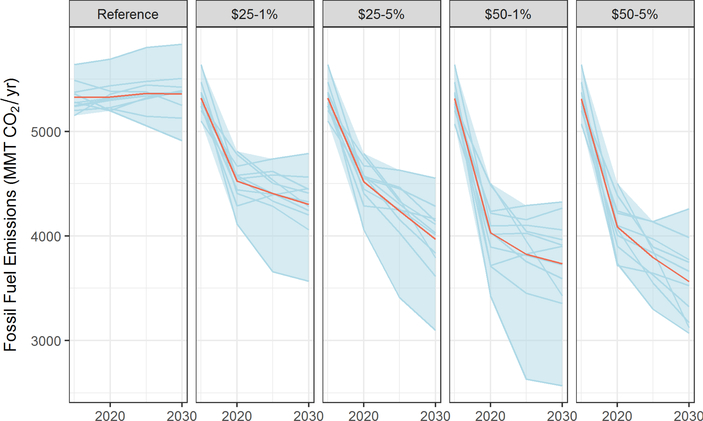
Annual fossil fuel emissions levels (MMt CO_2_) by year under the reference case and the four core carbon tax trajectories. For this and subsequent figures, the red lines show the average values across the models, the blue shaded area shows the range of model results, and the individual model trajectories appear in blue. For better readability, the vertical axis here does not start at zero. As mentioned in Sec. 1, model identity is generally not relevant to our conclusions here and so individual models are not identified. Note that the model with the most aggressive reductions did not report results for the $50–5% scenario, which explains the deeper maximum reductions in the $50–1% scenario.

Carbon dioxide emissions in the United States are projected to remain a significant contributor to rising CO_2_ levels in the absence of policy. As shown in Fig. 1, consistent with calibration to the EIA AEO 2016, the model projections of carbon dioxide emissions in the reference scenario are relatively flat, with limited changes from today’s levels. In all figures of this kind in this paper, the red lines show the average values across the models, the blue shaded area shows the range of model results, and the individual model trajectories appear in blue.

All of the carbon pricing policy scenarios significantly lower emissions compared with the reference scenario; the larger the carbon price, the deeper the emissions reductions (Fig. 1). A carbon price of $25 in 2020 that rises at 1% per year reduces CO_2_ emissions roughly 16–28% below 2005 CO_2_ emissions levels^6^ by 2020 and 17–38% below 2005 levels by 2030. A carbon price of $50 in 2020 rising at 5% per year reduces emissions 21–35% below 2005 levels by 2020 and 26–47% below 2005 levels by 2030.^7^

As shown in Fig. 1, the impact of the rate of increase in the carbon price above the rate of inflation is modest over the short run. A $25/ton tax rising at 5% only reduces emissions 19% more by 2030 than a $25/ton tax rising at 1%. The escalation rate matters more over longer time scales, as discussed below.

The variation in projected emissions reductions across multiple models in Fig. 1 highlights the uncertainties inherent in the emissions outcome of a carbon tax approach. Particularly closer to 2030, the models differ markedly in their projections of emissions. As noted in Sec. 2, variations across models do not capture the full range of uncertainty in potential outcomes, which also depend on factors such as technology costs and economic growth. This suggests that achieving a specific national emissions target (annual or cumulative) could require policymakers to adjust tax rates periodically (Burtraw and Palmer, 2013) or incorporate into the policy an automatic adjustment to the tax rate or its rate of growth (Aldy, 2017; Hafstead *et al*., 2017; Murray *et al*., 2017). Important policy design considerations arise in different approaches to adjusting tax rates over time, but a full discussion of these is outside the scope of this study.

Because CO_2_’s effect in the atmosphere is long-lived and climate forcing (the build-up of heat in the earth’s atmosphere and oceans) scales with the total CO_2_ concentration in the atmosphere, cumulative emissions are a better metric of the environmental impact than emissions in any one year. The range of cumulative reductions from 2020 to 2030 overlaps across all four core price trajectories, although the $50 trajectories achieve 50% more cumulative reductions on average or roughly 10% points (i.e., 20% versus 30%) more than the $25 trajectores.^8^ A higher escalation rate has a smaller impact compared to starting price over the first decade, with the $50/ ton tax rising at 1% achieving roughly 40% more total emissions reductions than the $25/ton tax rising at 5% (Table 1).

In the outer years (2038–2050), the carbon price for the $25–5% scenario exceeds that in the $50–1% scenario so that, extending the results in Table 1, the cumulative CO_2_ emissions reduction of the two policies is roughly equivalent over the 2015–2050 time frame. There may be policy reasons (e.g., reducing initial price shocks) to favor a lower starting point and higher growth rate, but it comes with the tradeoff of lower emissions reductions in the first decade of the policy. This reduces the flexibility to achieve deeper overall reductions if new information increases the projections of the risk from climate change, even while it may improve the policy’s short run political sustainability.

**Figure 2.**
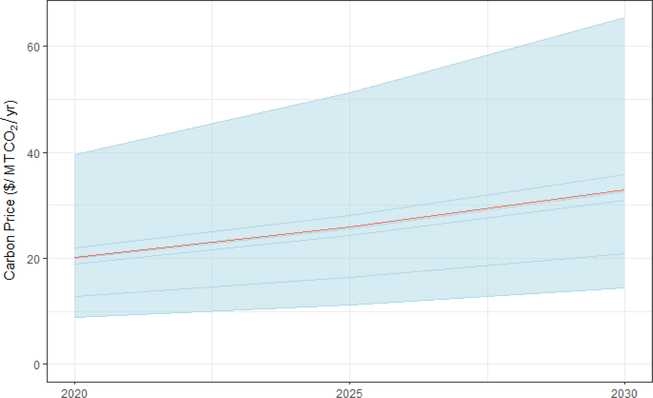
Carbon price trajectories that achieve a roughly 26% reduction in CO_2_ emissions in 2025 relative to 2005 levels. All models assumed a 5% escalation rate per year in the tax.

All of the four core price trajectories appear sufficient to achieve (and often exceed) a 26% economy-wide reduction below 2005 levels by 2025 (Fig. S1), at least with regard to the fossil-fuel-related CO_2_ component of the target. This target represents the less stringent end of the U.S. Nationally Determined Contribution to the Paris Agreement (26–28%) (UNFCCC, 2016). While the Trump administration has announced its intention to withdraw from the Paris agreement in 2020 (the first year withdrawal is feasible), this target remains a focus for some states and mayors (Gustin, 2017). Models found that a price trajectory to achieve the 26% reduction target would generally begin between $9 and $40/ton in 2020 and rise to between $11 and $51/ton by 2025 (Fig. 2).^9^ This scenario assumed a real annual escalation rate of 5%, which gives all of the trajectories a similar shape.^10^

All of the core carbon price trajectories modeled in this study achieved considerably more emissions reductions than would likely have been achieved under the Clean Power Plan. This result is not surprising as the prices required to achieve the goals of the Clean Power Plan range from modest to zero depending upon the technology assumptions and model. Additionally, although power sector reductions dominate the abatement in the scenarios here, reductions from other sectors can also be important in the short run and become essential for long-term targets with deep reductions (see Sec. 6).

Many models demonstrate close competition between natural gas and renewables. All else equal, lower natural gas prices will lead to higher emissions while cheaper renewables will lead to lower emissions. Analysis in the power sector suggests that low energy efficiency costs are also effective at reducing emissions, but only if the price of natural gas is not too low (Jaffe and Stavins, 1995). Ultimately, observed emissions reductions follow from the interaction of the tax policy with all other policy changes over the time window including other taxes and tax credits, other policies that impact the price of energy and energy technology, and any policies that effect overall economic growth.

Importantly, for a given carbon tax path, the different uses of revenue appear to have little, if any, impact on emissions. This gives policymakers flexibility to address other policy concerns with revenue without sacrificing environmental outcomes. However, if the policy is not revenue neutral, this principle may not hold. For example, if the revenue were used to purchase additional GHG reduction measures (for example, by funding reductions in non-CO_2_ GHGs), that would lead to greater overall emissions reductions. Using the revenue to blunt the price signal to consumers (for example, by subsidizing electricity purchases in proportion to use) could undermine emissions abatement by reducing the degree to which the price reduces demand.

### 3.2. Reductions in conventional air pollutants

Fossil fuel use generates pollution other than CO_2_, including from mining and oil and gas production (Epstein *et al*., 2011; Alvarez *et al*., 2012; Lutz *et al*., 2013), fuel handling (Jha and Muller, 2017), combustion emissions (National Research Council, 2010; Muller *et al*., 2011; Shindell, 2015), and waste disposal (Lemly and Skorupa, 2012; Lemly, 2015). Thus, when carbon taxes reduce fossil fuel use, especially from coal and transportation fuels, they also reduce those other non-GHG pollutants and can generate significant additional social benefits (a.k.a. co-benefits).

The potential co-benefits of reductions in particulate matter (i.e., PM_2.5_), nitrogen oxides (NO_x_), and sulfur dioxide (SO_2_) have been especially well documented. Sulfur dioxide and nitrogen oxides are air pollutants that harm human health and the environment and derive in part from fossil fuel combustion. Accordingly, emissions of both (which are also precursors to PM) are likely to decline under a carbon tax. These emissions reductions are especially notable because they are often significant in terms of magnitude (they can be twice the percentage reduction of CO_2_), the resulting health benefits can be significant on a macroeconomic scale, and the reductions occur rapidly in the first decade of the policy — accruing benefits to current generations.

**Figure 3.**
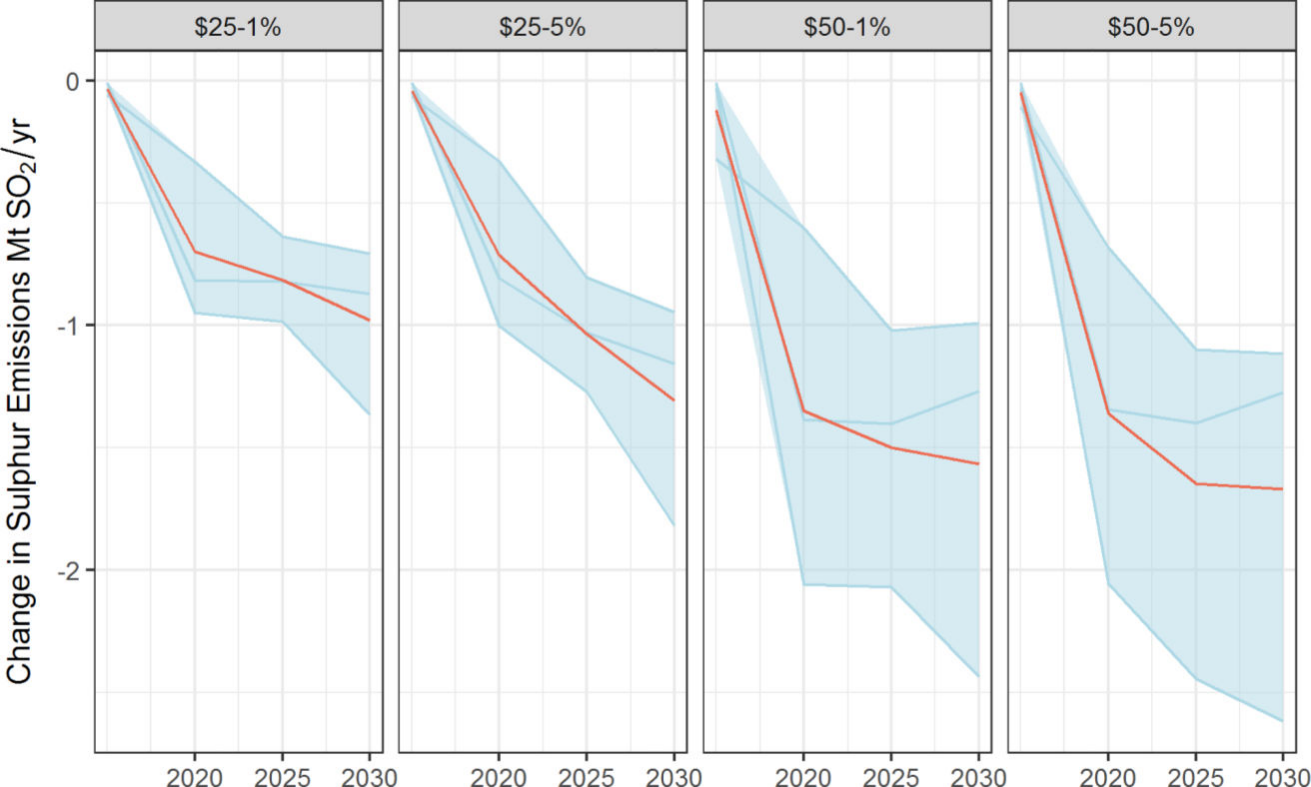
Sulfur dioxide reductions from the electricity sector relative to reference (Mt SO_2_/year) by year under four carbon tax stringencies. Only three models reported this variable.

Three models in this study estimated emissions reductions for SO_2_ from the electricity sector (Fig. 3).^11^ They project that SO_2_ emissions will decline in the reference scenario (not shown) by roughly 25–60% by 2020, likely from a combination of ongoing shifts in fuel use and environmental regulations. As Fig. 3 shows, carbon taxes reduce SO_2_ emissions relative to that declining baseline by roughly an additional 1Mt under the $25 price trajectories (52–89% from reference) and 1–2Mt under the $50 price trajectories (85–100%). Unlike the benefits from reducing GHGs, which accrue in the later years of the policy, the benefits from reductions in SO_2_ begin as soon as the policy is implemented (in this case 2020), with the bulk of the reductions occurring in the first 5 years.

As seen in Fig. S2, the carbon tax also reduces NO_*x*_ emissions from the electricity sector, but by a smaller amount. NO_*x*_ emissions fall by roughly 0.5–1Mt/year under $25 tax trajectories and by roughly 0.5–1.5Mt/year under the $50 tax (Fig. S2). We expect NO_*x*_ to show a smaller decline than SO_2_ as both coal and natural gas combustion generate NO_*x*_, whereas only coal produces SO_2_.

Studies of CO_2_ abatement policies often estimate substantial health benefits from consequent reductions in SO_2_ and NO_*x*_, frequently of magnitude similar to the near-term costs of the policy (Nemet *et al*., 2010; Burtraw and Palmer, 2013; West *et al*., 2013; Thompson *et al*., 2014; Buonocore *et al*., 2016). Accordingly, some studies suggest that considering co-benefits warrants more stringent climate policy than would a focus only on GHG benefits (Nemet *et al*., 2010; Boyce and Pastor, 2013).

Consistent with this literature, we find here that the emissions reductions from the electricity sector alone imply significant health benefits. Using EPA’s Co-benefits Risk Assessment (COBRA) tool (Abt, 2017), we estimate that the projected health effects for the average reduction in SO_2_ and NO_*x*_ in 2025 from a $25 carbon tax are on the order of 3,500–8,000 avoided cases of premature mortality and 90,000 cases of exacerbated asthma. This corresponds roughly to a monetized value of $31–71 billion in health benefits (3% discount rate) using standard epidemiological estimates (Krewski *et al*., 2009; Lepeule *et al*., 2012) and EPA methods, with the bulk of the benefits accruing in the upper Midwest and East Coast (Fig. S3). These estimates only reflect health outcomes for which U.S. federal agencies typically monetize health benefits (e.g., premature mortality); they do not include other harder-to-quantify and monetize health benefits or economic benefits such as increased labor productivity/participation. Emissions reductions from other sectors or other pollutants would further increase health benefits.

While most models do not report economy-wide changes in co-pollutants, Woollacott (this issue) estimated economy-wide emissions of SO_2_ and NO_*x*_ and benefits by tracking changes in fuels and found that more than half of the marginal welfare costs of the policy are offset by the subset of co-benefits considered. Specifically, the study estimated that a carbon tax of $25 per ton rising at 5% per year would avert 8,600–19,300 cases of premature mortality worth $72–162 billion in total monetized health effects based on the present value of emissions reductions over the policy period 2020–2040 ($2016). They estimate that average co-benefit varies by U.S. region and is approximately $150–1,250 per household.

## 4. Outcomes Over the Long Run

This study can inform the near-term design framework of a carbon tax in the United States, but it does not establish the carbon price path needed to hit any particular long-term emissions reduction target or estimate the associated economic implications of such goals. Long-term outcomes remain uncertain, meaning that policymakers should plan for updates to the policy to adjust toward their goals and account for new information, technology, and diplomatic developments.

Modelers ran all of the scenarios in this study over long time horizons, in most cases out to 2050 and beyond, but the fact that there is model output does not make the results robust for policymaking. These long horizons capture the full trajectory of a carbon price and are necessary to represent deployment of very long-lived capital, such as power plants. They also represent an important time scale for climate policy, as the climate will continue to warm unless global net emissions are reduced to near zero (or negative) in the longer term (IPCC, 2014). Some papers in this special issue and other studies of climate policies present results, including carbon prices, out to 2050. This is appropriate in a model inter-comparison research context. However, the uncertainties in such long run projections strongly limit their usefulness in policy analysis (Rosen and Guenther, 2015). Accordingly, we focus on reporting the policy-relevant short-term results (i.e., up to 2030) in this paper as our assessment is that the uncertainties are too large to make the post-2030 results useful for near-term policy design.

At least two analytical challenges become much stronger over longer time scales. First, as noted in Sec. 2, model parameters based on current technologies and historical relationships are likely to be increasingly obsolete over longer time scales and limit modelers’ abilities to project deeper, long-term de-carbonization. Important changes can include new technologies, such as new kinds of energy storage, or societal or cost shifts that significantly alter how actors can lower emissions. In many cases, the responsiveness of a sector to a carbon price in a model is based on past relationships between commodity prices and fuel use, for example, relating changes in the oil price to oil consumption, that may change greatly in the long run. If electric vehicles or advanced biofuels gain market share, or if autonomous vehicles and ride sharing change patterns of vehicle usage, then oil use in the transportation sector may become much more responsive to a carbon price than models currently project. Economic conditions, such as population growth and mobility, can also shift in ways that significantly alter the impacts of a policy.

Secondly, while some models include significant detail about current abatement opportunities in the electricity sector, their detail in other sectors is quite limited (Kaufman *et al*., 2016). This potentially constrains how well they can capture abatement that would be observed in the real world, especially in later years. For example, once the power sector is largely de-carbonized, electrification can significantly reduce emissions in the other sectors. However, none of the models in this study demonstrated significant electrification of other sectors, even in the most ambitious carbon tax scenarios. For instance, models did not report electric vehicle deployment, and none showed large shifts in emissions from transportation — suggesting limited electrification despite policy developments that steer markets in that direction.^12^

These limitations are illustrated in the results for carbon tax trajectories that achieve a 26% reduction in 2025 and an 80% reduction in 2050. Models generally found carbon tax paths starting around $20/ton but rising rapidly to very high levels in the long run (both starting points and escalation rates varied). However, these price trajectories likely say more about the difficulties the models have achieving these targets than the actual prices that will be needed. Indeed, several models in the study were unable to solve for price paths that hit the long-term target. For the models that did present these analyses, many had significantly reduced coal-based generation and emissions by 2030/2035 and seemed to find limited opportunities for technology shifts to reduce emissions after that. After 2030, the emissions reductions from the $50/ton tax rising at 5% a year are similar to reductions achieved by much lower tax trajectories. This suggests that most of the reductions in the out years of these models do not come from technology deployment (electrification, new processes, or technologies) but simply from the model raising prices to the point where demand is reduced (Tavoni and Tol, 2010; Barker and Crawford-Brown, 2013). As noted earlier, the introductions of new technology or shifts in behavior could mean that, in practice, lower carbon prices achieve the needed reductions. Similarly, the anticipation of high carbon prices could lead to investment in research and development or other shifts in investment dynamics that are poorly represented in the current models.

Because this project is best suited to informing nearer-term policy design issues, we recommend that policymakers look to different modeling tools for insights beyond the current horizon. Some studies, such as the Deep Decarbonization Pathways Project and the U.S. Midcentury Strategy (Williams *et al*., 2014; Bataille *et al*., 2016; The White House, 2016), have examined deep de-carbonization goals in the midcentury time frame. They use technology-rich integrated assessment models to explore uncertainties related to the evolution of technologies, economic conditions, and social dynamics over the coming decades and attempt to identify findings that are robust across scenarios and relevant for policymakers. However, owing to limitations similar to those described earlier, these studies have not attempted to estimate carbon prices that achieve the deep de-carbonization scenarios they examine.

## 5. Economic Outcomes

Economic evidence shows that a carbon tax, or emissions pricing more generally, is the most cost-effective way to reduce carbon dioxide emissions from the combustion of fossil fuels (Goulder and Hafstead, 2018). However, the economic outcomes may depend on several key design dimensions, including the price trajectory and the use of the revenues. This section focuses on two particularly salient outcomes: the amount of revenue raised and the macroeconomic outcomes (as measured by GDP or welfare).

### 5.1. Revenues

**Figure 4.**
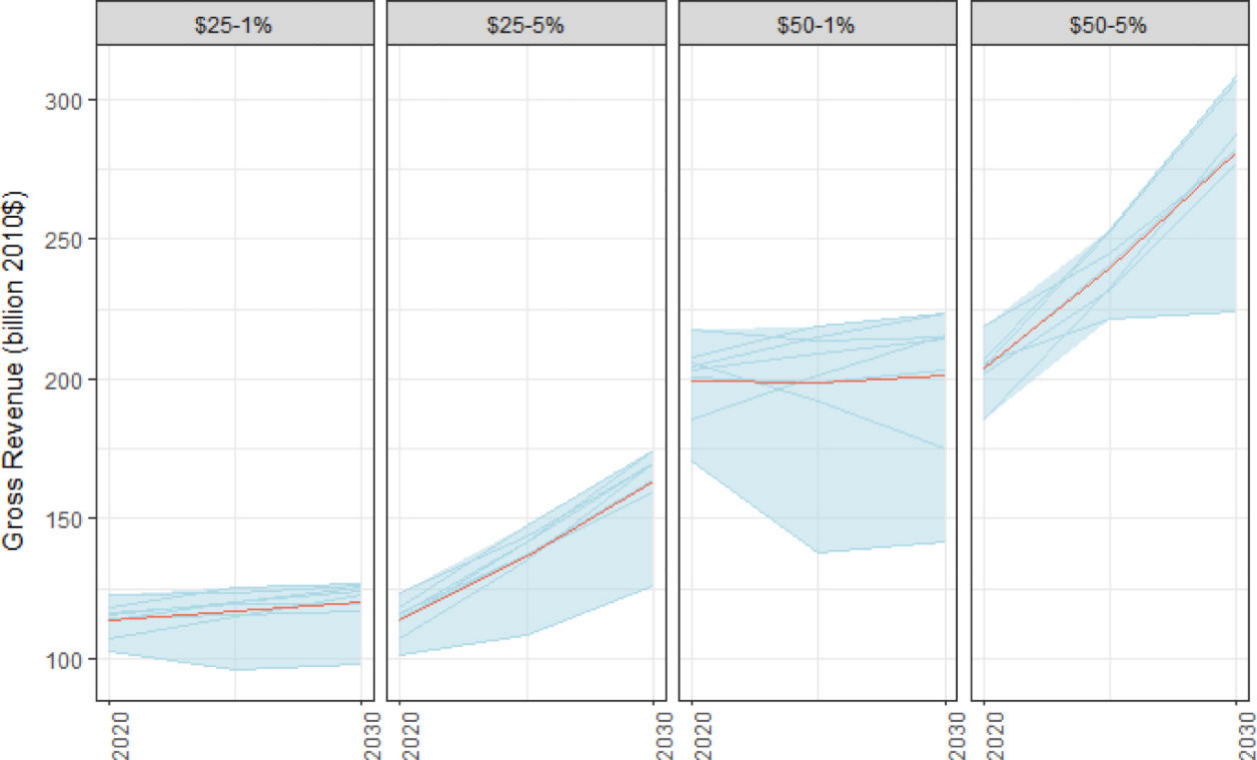
Annual gross revenue from carbon tax by tax rate trajectory. Household rebate scenarios.^14^

Carbon taxes raise significant gross revenues, i.e., the total revenue without accounting for policy-induced decreases in other federal revenues. Figure 4 displays the level of gross carbon revenues across the four core tax scenarios.^13^ For each tax trajectory, the differences in revenue across models correspond directly to their differences in projected emissions, as shown in Fig. 1.

Estimated annual revenues at the outset of the policy are relatively consistent across models, about $110 billion under a $25 tax and $200 billion under a $50 tax. As expected, models that predict less emissions abatement as a result of the carbon price predict more revenues from taxing those emissions. Most models report increasing revenues over time in the scenarios with 5% real growth in the carbon tax, despite decreasing emissions, because the real increase in the tax rate dominates the decline of the tax base. Revenues rise more modestly in the scenarios with 1% annual real growth in the carbon tax rate and decline in some cases.

Table 2 illustrates the variation in estimated revenue across models by tax scenario. It displays the minimum, average, and maximum levels of estimated gross revenues raised by the carbon tax over the 10-year window from 2021 to 2030. On average across the models, a $25/ton price rising at 5%/year would generate over $1.4 trillion. Even under the lowest estimate from any model at this price trajectory, carbon taxes will generate significant revenue, roughly $1.1 trillion.

To be clear, the gross revenue numbers in Table 2 are not equivalent to a 10-year budget score that the Congressional Budget Office (CBO) and the Joint Committee on Taxation (JCT) would produce. First, numbers in Table 2 are real values in 2010 dollars, accounting for projected inflation, whereas CBO and JCT score revenues in nominal terms.^15^ Secondly, the values in Table 2 do not account for the negative impacts of a carbon tax on other federal revenues, an adjustment known as the “haircut.” In their scores of excise tax proposals, CBO and JCT generally apply a haircut of about 25% to gross revenue estimates to account for decreased noncarbon tax revenues and increases in government costs (government spending on energy and social safety net payments (some of which are indexed to price levels)). McFarland *et al*. (this issue) report a measure of the haircut from the model results and find the results vary considerably due to differences in the models’ representation of different taxes and response of labor and capital supply to changes in tax rates. Due to these considerable differences, we recommend that policymakers defer to the official scores for estimates of the haircuts at this time.

While the projected revenues of a carbon tax are significant, they are relatively small in the big picture of federal public finance. For example, $100 billion (the amount roughly expected for a $25/ton tax in 2020) was 3% of all federal revenue in 2015. In the same year, the budget deficit was $439 billion, and the corporate income tax raised $344 billion. For comparison to existing tax expenditures that could be revised in a tax reform package, the home mortgage interest deduction costs an average of $120 billion annually from 2013 to 2017, and the tax deduction for employer payments for health insurance costs an average of $337 billion annually over the same period (Aldy, 2017). Also for comparison, the gross revenue from a carbon tax over the next decade is roughly similar to the size of the projected deficit impact of the tax legislation that was enacted in late 2017 (e.g., roughly $1 to 1.5 trillion, depending on the impact of economic growth) (JCT, 2017). As noted in Sec. 1, we hold government spending on everything (including interest payments) to baseline levels. In other words, the scenarios are all deficit neutral. Other carbon tax policy proposals could potentially retain some revenue for additional government spending.

### 5.2. Macroeconomic outcomes

**Figure 5.**
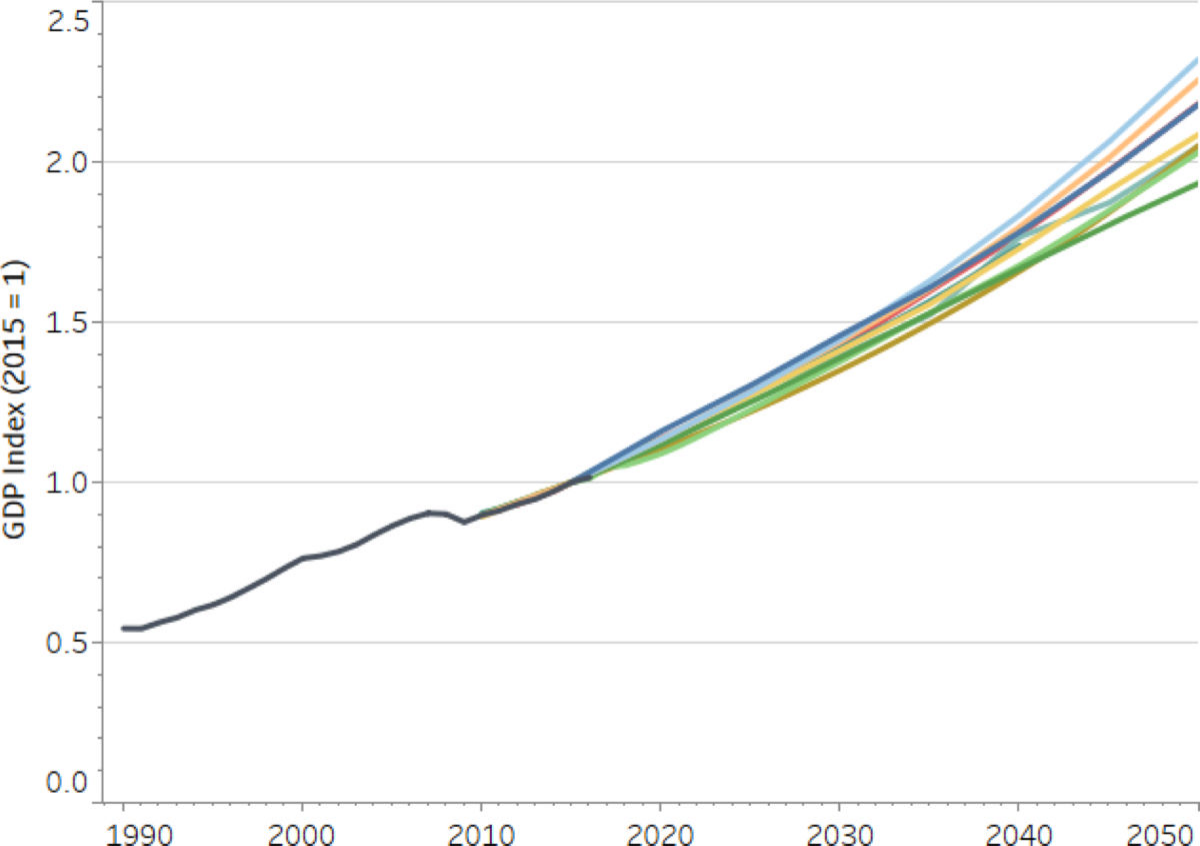
Historical GDP and projected GDP growth under the reference case. GDP in 2015 is indexed to 1 to allow comparison across models. These growth rates do not reflect economic damages that will occur from climate impacts or pollution in a reference case.

**Figure 6.**
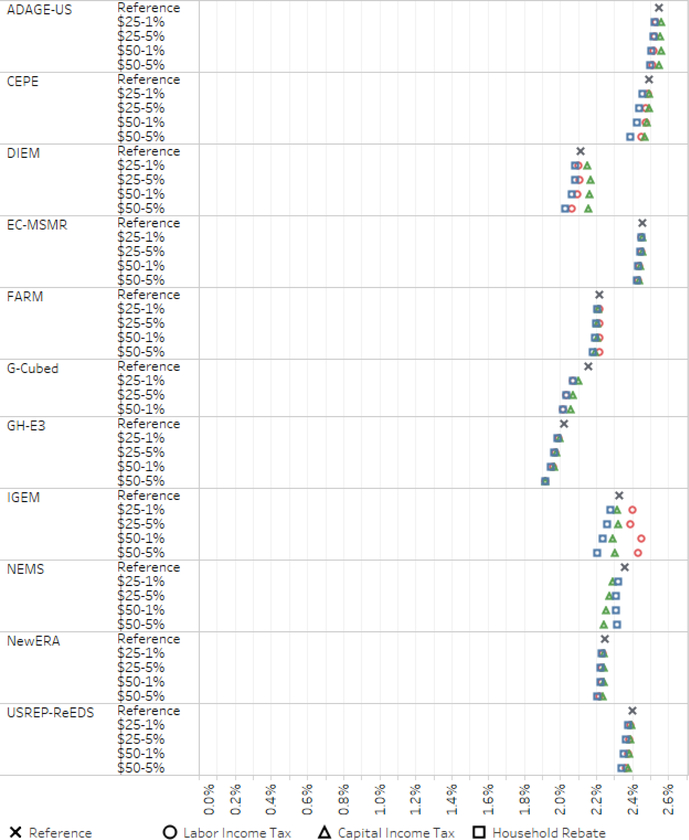
Rate of GDP growth. Growth in market exchange rate of gross domestic product across the four carbon price trajectories and three revenue recycling methods by model (2015–2030).

All models in the study project smooth growth in GDP into the future. Figure 5 displays historical GDP along with GDP reference projections in the different models in the study. Although modelers endeavored to harmonize their reference scenarios, differences remain. Thus, we normalize GDP levels in 2015 across models and display the GDP growth from 2015 to 2030. Variability in growth rates can be seen in Fig. 6.

Carbon taxes affect the economy directly by increasing the cost of fossil fuels and indirectly through the ripple effects across the economy. A carbon tax also affects the economy through fiscal policy channels. For example, by lowering returns from working and investing, a carbon tax can increase the economic burdens of preexisting labor and capital taxes — what economists refer to as the *tax interaction effect*. At the same time, how the federal government uses the revenue has economic implications. For example, using the carbon tax revenue to decrease other tax rates can create a *revenue recycling effect*, a decrease in economic burden from those other taxes. The size of the revenue recycling effect will depend on which tax is being lowered. If the revenue recycling approach more than offsets the overall costs of the policy, it creates a *double dividend*: a decrease in emissions accompanied by increased economic activity.^16^

A comparison of results yields a set of consistent findings across models. First, the economic costs, as measured by either GDP or welfare, are expected to be modest: annual GDP growth continues and the reduction in the level of GDP in future years relative to a business as usual case is projected to be below 1% even under substantial carbon tax levels. Secondly, the type of revenue recycling (through household rebates, labor tax cuts, or capital tax cuts) impacts the estimated economic costs of carbon taxation: cutting labor or capital taxes lowers the economic costs relative to household rebates.

Some findings vary substantially across models. For example, the size of the revenue recycling effect varies across models: some models generate small differences across revenue-recycling scenarios, whereas others generate a double dividend in some scenarios but not others.

Relative to total projected GDP, any delay in the growth of the size of the US economy due to the carbon tax is small. In the reference scenario (with no carbon tax or climate impacts), the U.S. economy reaches $25 trillion roughly in year 2034; under the carbon tax, the economy would reach the same level in the following year.

Figure 6 shows average GDP growth rates from 2015 to 2030 in both the reference and core policy scenarios for each of the models.^17^ The spread in GDP growth rates is considerably larger across models than across the scenarios within a model.

Some models and scenarios report lower GDP growth in the policy scenarios than the reference scenario and some report higher growth. However, these GDP impacts are small — equivalent to a change in the rate of GDP growth of less than 0.05% points in most models. Comparing across recycling options, the labor and capital tax cut scenarios result in lower GDP impacts than household rebates in all but one of the models (see McFarland *et al*., 2018). The differences in GDP outcomes across recycling options vary across models and are generally small. In some cases, the GDP effect is positive: a carbon tax combined with capital (or in some cases labor) tax cuts leads to higher economic activity. The attainment of the double dividend, however, is not robust across models. The existence of the double dividend within economic models depends largely on model structure related to including multiple inefficiencies and assumptions about how the supply of labor and capital respond to the carbon tax. A detailed examination of the technical conditions in which models achieve the double dividend is beyond the scope of this paper.

An alternative, broader measure of economic outcomes is household welfare (Paltsev and Capros, 2013). In the EMF 32 models, welfare changes estimate the dollar-equivalent change in household utility that the policies create via changes in prices, wages, and returns to capital. Welfare also includes factors that are not included in GDP, particularly leisure.^18^ For example, if households work more than they otherwise would in response to lower labor tax rates, the measure of welfare accounts for their loss of leisure, whereas GDP would not. These estimates do not include welfare benefits of reduced conventional pollution or climatic damages.

**Figure 7.**
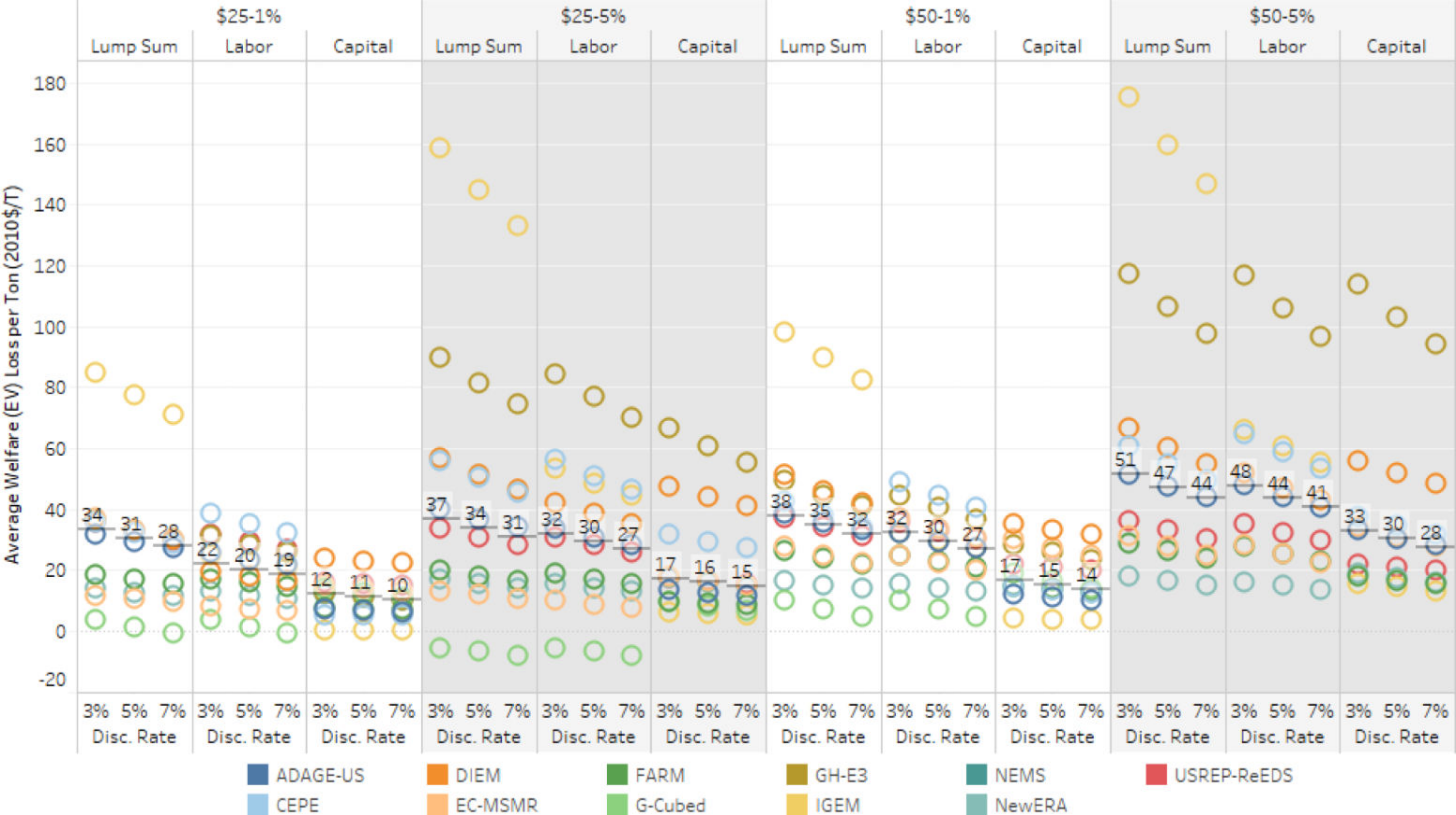
Average welfare loss per ton of CO_2_ emissions reduced (relative to reference) from 2020 to 2030 in the core tax scenarios under three discount rates. Median welfare across all models is marked with a line and label for each discount rate and policy combination. Welfare does not reflect health benefits or reduced climate damages resulting from the carbon price. Each color represents a different model.

We see in Fig. 7 that the welfare rank order of recycling options is generally constant across models and discount rates. Household rebates are consistently the (macroeconomically) most costly revenue option; labor and capital tax cuts induce lower welfare costs, but the size of their relative differences varies. Fewer models achieve a double dividend for welfare than GDP. The differences, while difficult to diagnose, largely stem from differences in the structure of taxes and the responsiveness (elasticity) of labor supply across models.

To examine the range of welfare outcomes, let us consider outcomes in a particular year. In 2025, the one-year welfare costs of the $25–5% price path with rebates vary from about $5 per ton reduced to about $140 per ton reduced. Under capital and labor tax cuts, the spreads of welfare costs per ton reduced are $5 to $60 and $5 to $75, respectively. In each of the three recycling scenarios, the median welfare cost per ton reduced is less than $40.

In practice, these gross welfare costs per ton abated would be offset by the net benefits derived from reducing CO_2_ and co-pollutants. Applying an estimate of the global benefits of CO_2_ reductions (a.k.a. a social cost of carbon, or SCC) in 2025 of, for example, $48 per ton ($2010), global climate benefits exceed the domestic policy welfare costs in a majority of the models across all recycling options.^19^ Evaluating only domestic benefits of reductions in 2025 at about $7 per ton, consistent with the recently revised U.S. government approach, would show fewer scenarios with net benefits.^20^ These estimates do not account for changes in other GHGs, such as methane, which could occur with a carbon tax, or the benefits of lower conventional air pollution. For example, the monetized benefits from domestic SO_2_ and NO*x* reductions are roughly on the order of $30–65/ton of CO_2_ reduced in 2025 (see Sec. 3.2).

A full discussion of a global versus domestic SCC estimate is beyond the scope of this paper; however, some recent literature may provide useful context for the reader. The National Academy of Sciences review of the SCC noted that a domestic estimate does not capture climate impacts in other countries that also have impacts on the U.S. in a connected global economy.^21^ Kotchen (2016) summarizes the literature on global versus domestic SCC values and identifies conditions under which a country’s decision to internalize the global SCC would be individually rational.^22^ Other recent papers argue that the use of a global SCC would encourage reciprocal actions by other nations that would then benefit the United States and more generally promote more ambitious climate action (Guivarch *et al*., 2016; Revesz *et al*., 2017a, 2017b). A recent survey of economists (Howard and Sylvan, 2015) suggested strong support for values on the order of $40/ton or greater. However, others have argued for the use of a domestic SCC, arguing that benefit–cost analysis of unilateral action by one country should focus on the domestic benefits and costs (Fraas *et al*., 2016; Gayer and Viscusi, 2016, 2017). There are also important economic and ethical considerations beyond our scope here associated with the selection of the discount rate (National Academies of Sciences, Engineering, and Medicine, 2017).

## 6. Distributional Outcomes

The welfare outcomes described in Sec. 5 are aggregate (or economy-wide) outcomes, but individual households will be affected differently (Williams *et al*., 2015; Cronin *et al*., 2017; Fremsted and Paul, 2017). The distribution of costs depends on each household’s carbon intensity of expenditures and source of income. If low-income households spend a larger fraction of their income on electricity or gasoline, the burden of higher energy prices as a share of income will be higher for these households. If households with high levels of education do not work in energy-intensive industries, the income effect of lower labor demand in those sectors will be lower as a share of income for these households. Additionally, the method of revenue recycling will impact the distribution of costs across households.^23^ Households that own capital (i.e., high-income households) will benefit proportionately more from reductions in capital taxes than households that do not own capital (i.e., low-income households).

We can analyze the economic outcomes of carbon taxes across a wide-range of economic and demographic dimensions: income, expenditure, wealth, age, education, state of residence, occupation, industry of employment, etc. Four models in this study evaluated the distribution of costs across five income quintiles. Average welfare costs, as a percent of full consumption, for households in each quintile of the income distribution demonstrate how costs vary across households and how recycling options impact the relative costs across households. Welfare estimates include changes in consumption and leisure, but do not include improvements in air quality or avoided climate damages.

**Figure 8.**
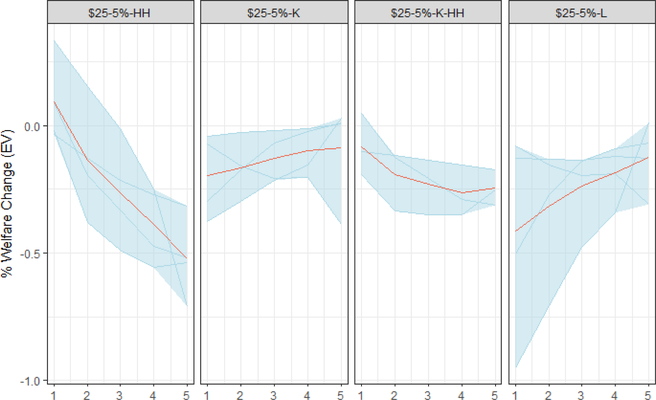
Change in welfare (equivalent variation) across four revenue recycling options by income quintile from 2020 to 2030, discounted at 3% for the $25–5% tax scenario. HH — lump-sum household rebate; K — capital tax; K-HH — 50/50 split between K and HH; L — labor tax cut. Welfare does not reflect health benefits or reduced climate damages resulting from the carbon price.

Figure 8 shows the cumulative welfare change for 2020–2030 across income quintiles for the $25–5% scenario under four revenue recycling options. The four revenue recycling options include the three options evaluated previously: lump-sum rebate to households (HH), capital tax reductions (K), and labor tax reduction (L). Additionally, a hybrid recycling scenario that combines recycling options is included: half capital tax reduction and half lump-sum rebate (K-HH). The distributional outcomes are a product of two inter-related features of the policies: their macroeconomic outcomes and the distribution of the revenue.

Lump-sum rebate — the least economically efficient recycling option as described in Sec. 5 — is the most progressive revenue option. Across all models, rebate scenarios provide more relative benefit to lower income households relatively than higher income households because the rebates represent a relatively larger share of their income and they are relatively unaffected by differences in macroeconomic outcomes. Whereas on average across models, these households show no change to a slight benefit (0.6%) from the rebate scenario, higher income households experience a 0.3–0.7% loss; they are more affected by lower macroeconomic performance and they do not benefit on net from the rebates. Relative to providing rebates, modeling suggests that reducing capital taxes (K) results in lower welfare for the lowest income households (0% to 0.3%) and in higher income for higher income households (0% to −0.4%). The hybrid recycling scheme, in which the carbon revenue is split between lump-sum rebates and capital tax reductions, is mildly progressive: the welfare change ranges from roughly 0% to 0.2% for the lowest quintile down to 0.2% to 0.3% for the highest quintile. Although this policy is less efficient than the full capital tax policy (K), the four models demonstrate that the burden is relatively evenly distributed across households under the mixed recycling policy.

In several of the models, recycling revenues to offset labor taxes is the most regressive of the four approaches. Lower income households receive a greater percentage of income from government transfers, and a reduction in taxes on labor has little effect on their income. Facing increased energy prices from the carbon tax and without lump-sum rebates to counteract these impacts, the welfare changes for lower income households range from −0.1% to −0.9%.^24^ This wide range in welfare impacts suggests that model structure and assumptions play a major role in the impact of a labor tax cut. The welfare loss for high income households under labor tax reductions is roughly the same as under capital tax reductions.

Though we present four recycling approaches here, a multitude of other recycling options may be used to address distributional effects. Within the context of EMF 32, Caron *et al*. (this issue) present results suggesting that welfare losses to the lowest income households can be mitigated with little efficiency loss. Additionally, while this analysis considers the distributional implications across income classes, distributional considerations within income classes (Cronin *et al*., 2017), across regions (Caron *et al*., this issue), urban and rural areas (Fremsted and Paul, 2017), age cohorts (Rausch and Yonezawa, 2018), and occupations may also be important to how policymakers choose to use the revenue.

A few of caveats to these results apply. First, not all models reported in Fig. 8 necessarily capture the full range of transfer income across the income quintiles. For example, to the extent that low-income households disproportionately receive income from social safety net programs whose payments are indexed to price levels, they may be relatively more protected from the carbon tax burden than the figure suggests (Cronin *et al*., 2017).

Secondly, these results only speak to one aspect of the distributional impacts of this policy; the environmental benefits of the policies (and their associated economic impacts) will be unevenly distributed across households and regions. Households in areas with poor air quality and those that are vulnerable to climatic damages are often also low-income and may benefit disproportionately from the environmental outcomes of a carbon tax. Thus, while we focus here on the distributional outcomes of the carbon tax and its revenue, one should be mindful of potentially different patterns of *net* distributional outcomes.

Finally, the analysis of the distributional of outcomes may be limited due to model structure. For example, the CGE models that reported distributional outcomes for EMF 32 assume full labor mobility (no firm- or sector-specific human capital or skills). The negative outcomes for certain demographic groups may be underestimated if workers cannot easily shift firms or sectors in the short run. However, it is not immediately clear whether the full labor mobility assumption biases the results toward greater progressivity or regressivity.

## 7. Sector-Specific Outcomes

### 7.1. Sectors

**Figure 9.**
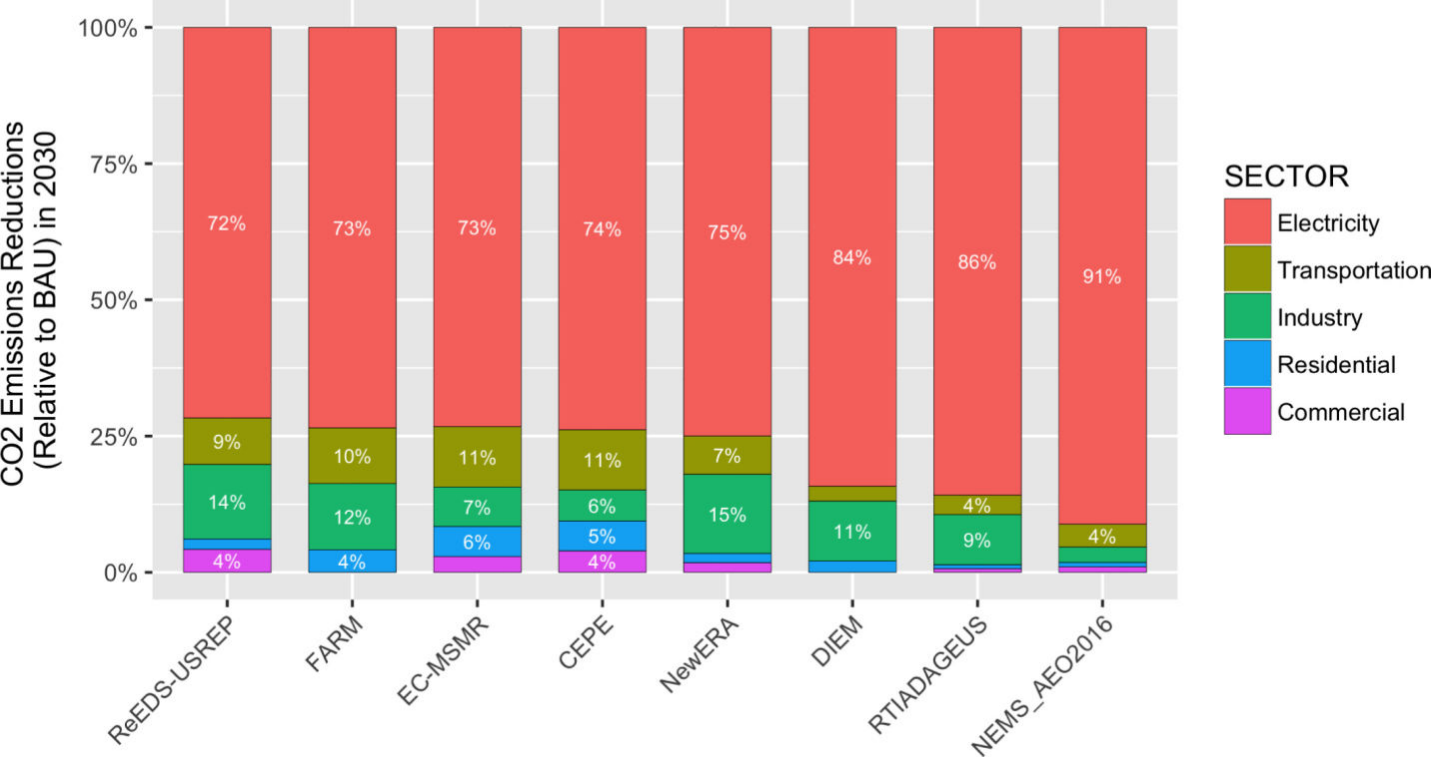
Percentage share of emissions reductions by sector. Illustrative results for the $25/ton tax rising at 5%/year in 2025. Only seven models report detailed sectoral breakdowns.

One of the chief advantages of an economy-wide carbon price is that it allows emissions reductions to come from the most cost-effective sources, regardless of sector. All of the models in this study clearly showed emissions reductions occurring primarily in the electricity sector (Fawcett *et al*., 2014; Kaufman *et al*., 2016). Across the four major carbon price pathways, 72–91% of the emissions reductions occurred in the electricity sector (Fig. 9). Models differed in which sector was the next most-responsive with some showing the second highest reductions from the industrial or transportation sector. Of the eight models reporting detailed sectoral breakdown, the version of NEMS used to generate AEO 2016 showed the least responsiveness in nonelectricity sectors.

**Figure 10.**
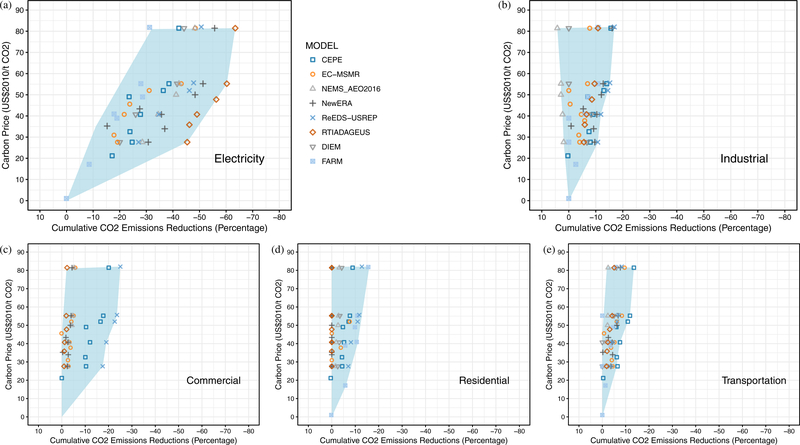
Cumulative CO2 emissions reductions (relative to reference 2015–2030) versus carbon price. Not all models reported detailed sectoral breakdowns.

As with the overall emissions reductions discussed earlier, there was considerable variability across models in the sector shares of emissions reductions for a given price (Fig. 10). The electricity sector was generally quite responsive, especially for prices up to $40–50/ton, whereas the residential and transportation sectors were the least responsive. The electricity sector would be expected to be responsive in the short term as economic dispatch of power plants and retirements provides relatively fast ways to achieve emissions reductions, as well as in the medium term as there are lower or zero carbon generation options that are cost-competitive with high carbon generation (especially with a carbon price in place). Residential housing and transportation, in contrast, both feature a very large stock of houses/cars that can be slow to turn over. Responses in other sectors tended to be more linear as prices changed.

The dominance of the electricity sector as a source of reductions does not, however, imply that a policy that covers only the electricity sector would be optimal. For example, a power-sector only policy will raise costs of electricity relative to other fuels, which can disadvantage electrification that would otherwise be beneficial for reducing carbon emissions (Dennis *et al*., 2016). A power-sector only policy could also encourage industrial facilities to run fossil power generation for local use — “escaping” the carbon price. Additionally, while the power sector is undoubtedly the most responsive sector to a carbon price, models often have limited representation of existing or future technologies that could drive reductions (e.g., electrification, energy efficiency opportunities, structural shifts) (Barron, 2018). As a result, the precise partitioning of reductions by sector may say more about the structure and assumptions of the model than what may happen in reality.

### 7.2. Fuels and energy consumption

**Figure 11.**
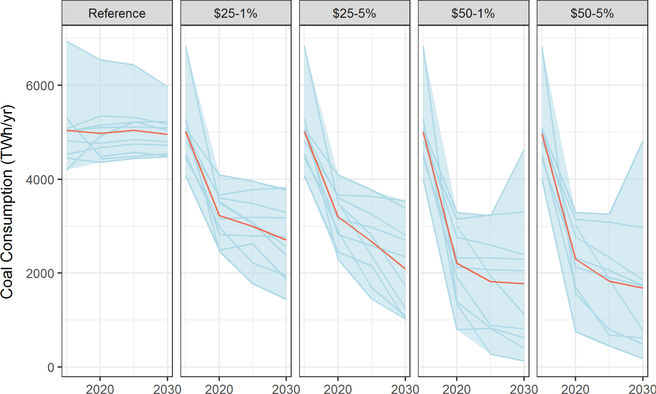
Coal consumption (TWh/year) under four carbon tax stringencies. Blue bands represent the range of model results, darker blue lines show the individual model results, and the red lines show the average value.

All of the price trajectories cause significant shifts in fossil fuel demand, with coal shifting the most significantly. All models robustly show coal consumption remaining roughly flat in the reference case. With the addition of a carbon price, coal consumption falls significantly (20–85%) as soon as the tax is implemented in 2020, with most models showing a gradual further decline from 2020 to 2030 (Fig. 11). In reality, the 2020 transition would not be so sharp as utilities are likely to phase power plant retirements to ensure reliability. Under the $50/ton price trajectory, coal demand falls to somewhere between 60% of reference levels and near zero by 2030, with the exception of one model (EC-MSMR) which shows significant carbon capture and storage (CCS) deployment in 2030 and associated increases in coal use.

**Figure 12.**
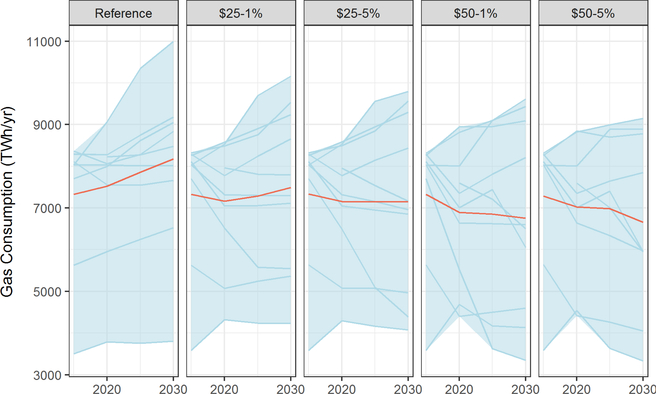
Natural gas consumption (TWh/year) under four carbon tax stringencies. Blue bands represent the range of model results, darker blue lines show the individual model results, and the red lines show the average value.

Natural gas, which has lower smokestack CO_2_ emissions per unit energy than coal, is projected to have roughly flat to rising consumption in the reference case (Fig. 12) — an aggregate effect from use in a number of sectors. A carbon tax has a mixed impact on natural gas demand in 2020 in all of the models, with natural gas use ranging from a decline of 31% to an increase of 24% above baseline levels. Natural gas use tends to be flat or falling toward 2030 at $50 carbon prices. Some models show relatively little change in gas use, whereas others show significant (40–60%) reductions in gas use, presumably by favoring renewables over gas.

Oil use in the U.S. economy sees the smallest changes of the three major fossil fuels (Fig. S4). The range of oil use across models is fairly broad, but with little downward trend in the average or individual model responses. As noted in Sec. 2, the historical calibration of responsiveness of the economy to oil price changes is a significant factor in this modest response.

**Figure 13.**
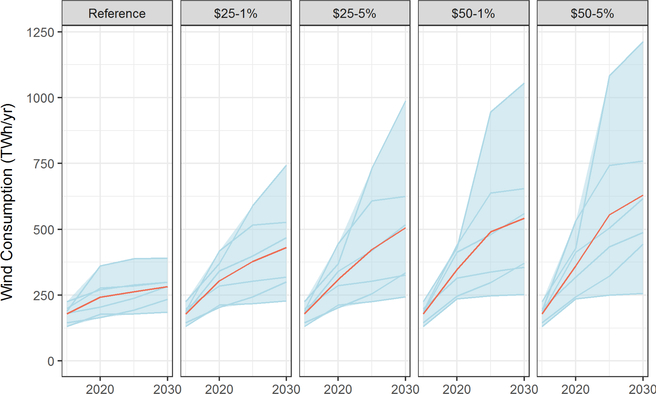
Wind energy (TWh/year) under four carbon tax stringencies. Blue bands represent the range of model results, darker blue lines show the individual model results, and the red lines show the average value.

**Figure 14.**
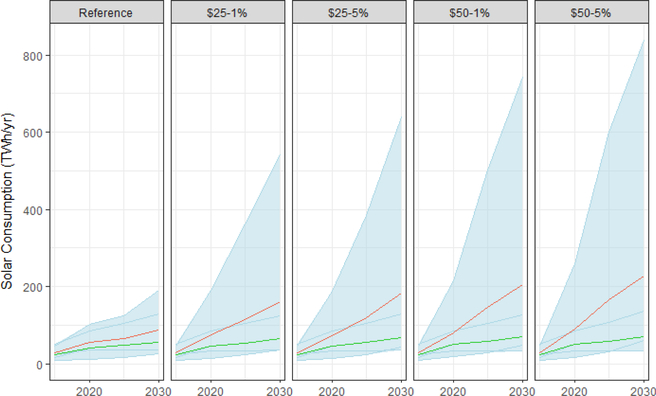
Solar energy (TWh/year) under four carbon tax stringencies. Blue bands represent the range of model results, darker blue lines show the individual model results, the red lines show the average value, and the green line the median.

Renewable energy shows a range of responses in the models. Zero carbon generation should be favored over fossil fuels but the details depend on technology cost assumption and details of the model. Virtually all models show increases in wind energy in the baseline, with significant increases above baseline levels under the carbon price scenarios (Fig. 13). By 2030, wind energy is 23–148% higher under a $25–1% tax and 48–300% higher under a $50–5% tax. In contrast, only one of the six models showed a significant increase in solar under the carbon tax scenarios (Fig. 14). These results flow directly from assumptions about the costs of solar relative to wind but seem unrealistic given the recent growth patterns in solar energy. It suggests that the models in this study may not be accurately capturing the current economics of solar, let alone how they may evolve in the future.

**Figure 15.**
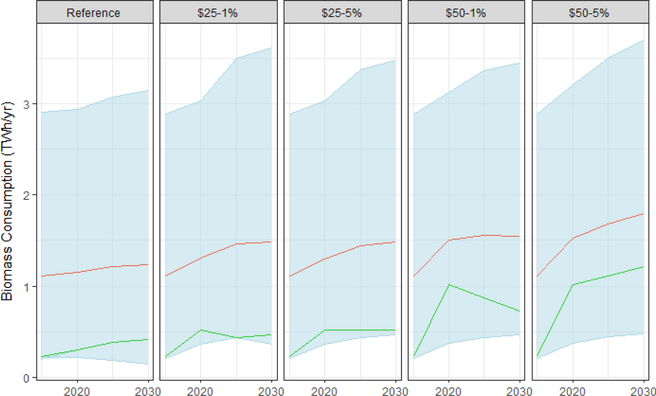
Biomass energy (TWh/year) under four carbon tax stringencies. Blue bands represent the range of model results, darker blue lines show the individual model results, the red lines show the average value, and the green line the median.

Results were similarly mixed for biomass with some models showing significant increases and others showing little change under a carbon tax (Fig. 15). For much more detail on renewable energy in the electricity sector, including results from more models and a greater exploration of technology costs, see Bistline *et al*. (2018) and Creason *et al*. (2018).

### 7.3. Prices

Energy price impacts are one of the most politically salient impacts of a carbon price. While increases in the price of fossil fuels are an inherent component of a carbon tax, the net effect on households depends upon how the revenue is recycled, whether energy efficiency or other technologies provide routes for households to reduce use of fossil fuels or goods and services whose prices are influenced by fossil fuel prices, and the benefits of the policy. In reviewing these price impacts, policymakers should keep in mind that the full economic impact on households is best measured by welfare after accounting for revenue recycling (discussed earlier).

**Figure 16.**
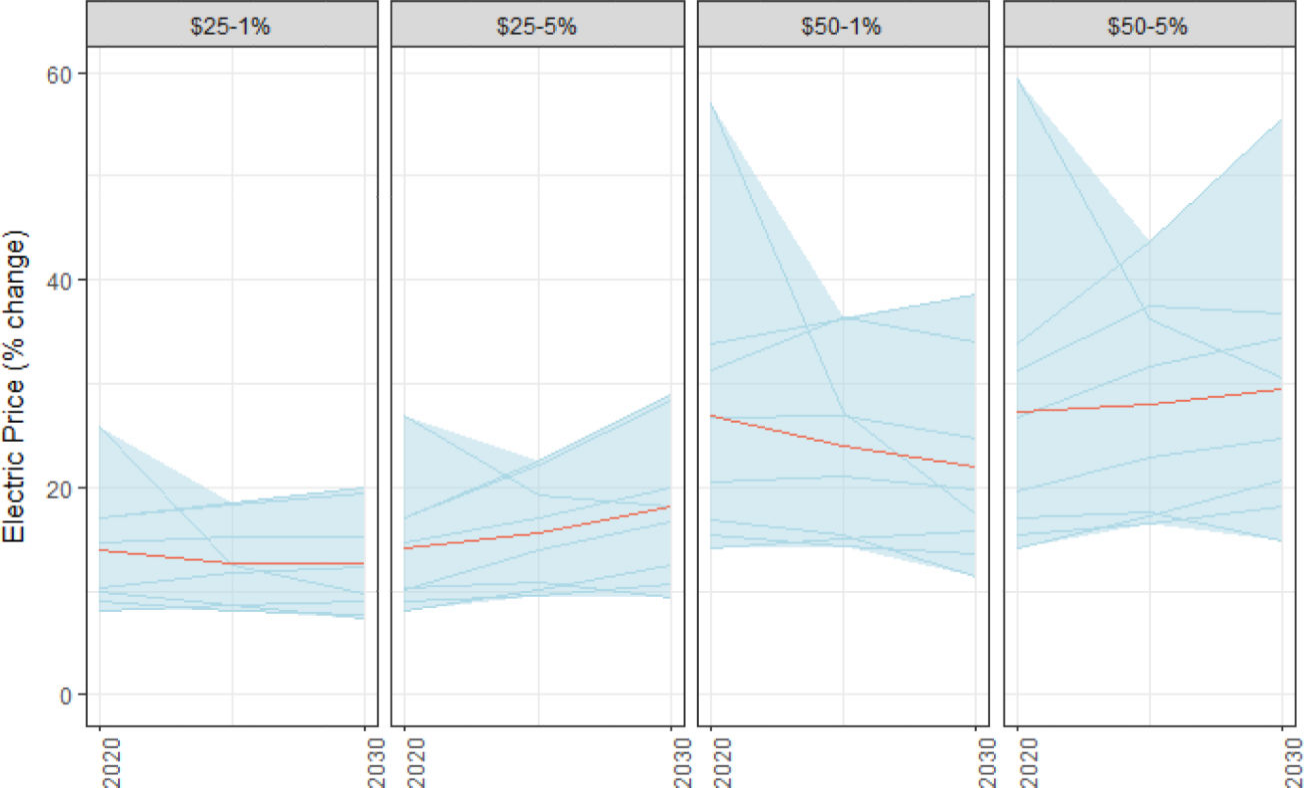
Electricity price under four core carbon tax paths (household rebate scenarios).

A carbon tax will increase electricity prices wherever fossil fuels set the marginal price of electricity. Electricity prices rise between 9% and 26% in 2020 under a $25/ton tax, while rising 15–59% under a $50/ton tax. By 2030, electricity prices rise 7–29% in 2020 under a $25/ton tax, while rising 23–56% under a $50/ton tax (Fig. 16). The wider range for a $50 tax suggests uncertainty about how cost-effectively the electric sector can decarbonize at that tax level. We represent these price increases as percentages, rather than cents/kWh because models differ somewhat in the degree to which they capture full retail electricity rates. Additionally, these price impacts will depend heavily on how well models capture existing trends toward lower carbon energy sources (Gilbert and Sovacool, 2016). To the extent that renewables deploy more quickly than anticipated in the reference case, the electricity price impact of the tax will be smaller (where renewables set the marginal price of electricity or reduce the need for high-cost fossil generation). These national price impacts obscure strong regional variability in the impact of a carbon price on electricity. Detailed results for electricity sector impacts are discussed in Bistline *et al*. (2018). In general, regions with more coal-intensive generating fleets today such as the Southeast and Midwest are likely to experience larger price increases (although those increases occur from electricity prices that are lower than many parts of the country like the Northeast that have already reduced the carbon intensity of the fleet over the last decade). Regions like the West Coast and the Northeast are likely to experience smaller increases. It is important to note that these are prices and that the actual costs are a function of usage (which can be reduced through energy efficiency policies) and only a component of overall energy spending. For example, electricity rates could increase but consumers who are relying on heat pumps or electric vehicles could still see their overall expenditures falling (Paltsev and Capros, 2013).

**Figure 17.**
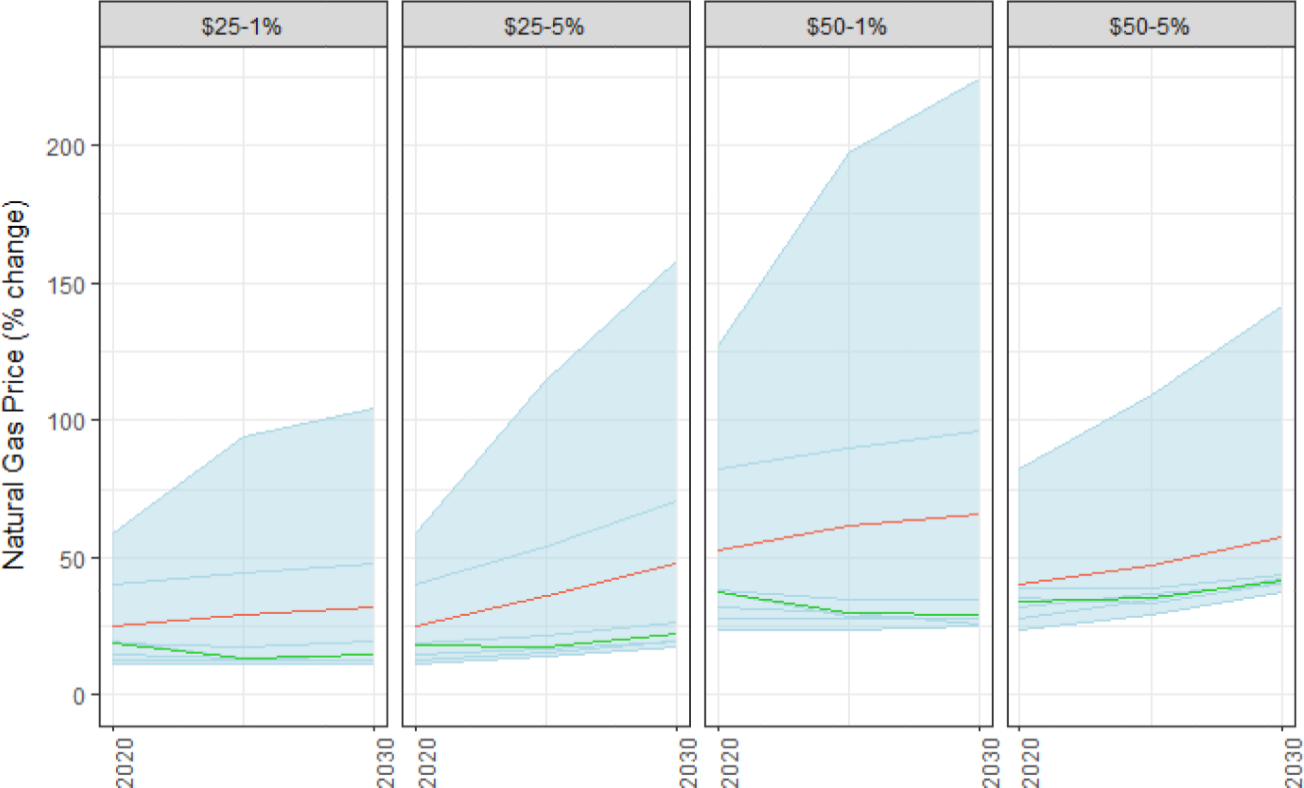
Percent change in residential and commercial natural gas prices. Blue bands represent the range of model results, darker blue lines show the individual model results, the red lines show the average value, and the green line the median.

Although natural gas usage varies significantly across models (Fig. 12), natural gas prices remain flat or increase as a result of the carbon tax (Fig. 17). Median natural gas prices increase 19% in 2020 under a $25/ton tax, while rising 34% under a $50/ton tax. By 2030, natural gas prices rise 26% under a $25/ton tax, while rising 42% under a $50/ton tax. A minority of the models show much higher increases in natural gas prices. Natural gas prices projections are strongly influenced by assumptions about natural gas supply and the economics of extraction; differences in the supply of natural gas can alter these price outcomes significantly.

**Figure 18.**
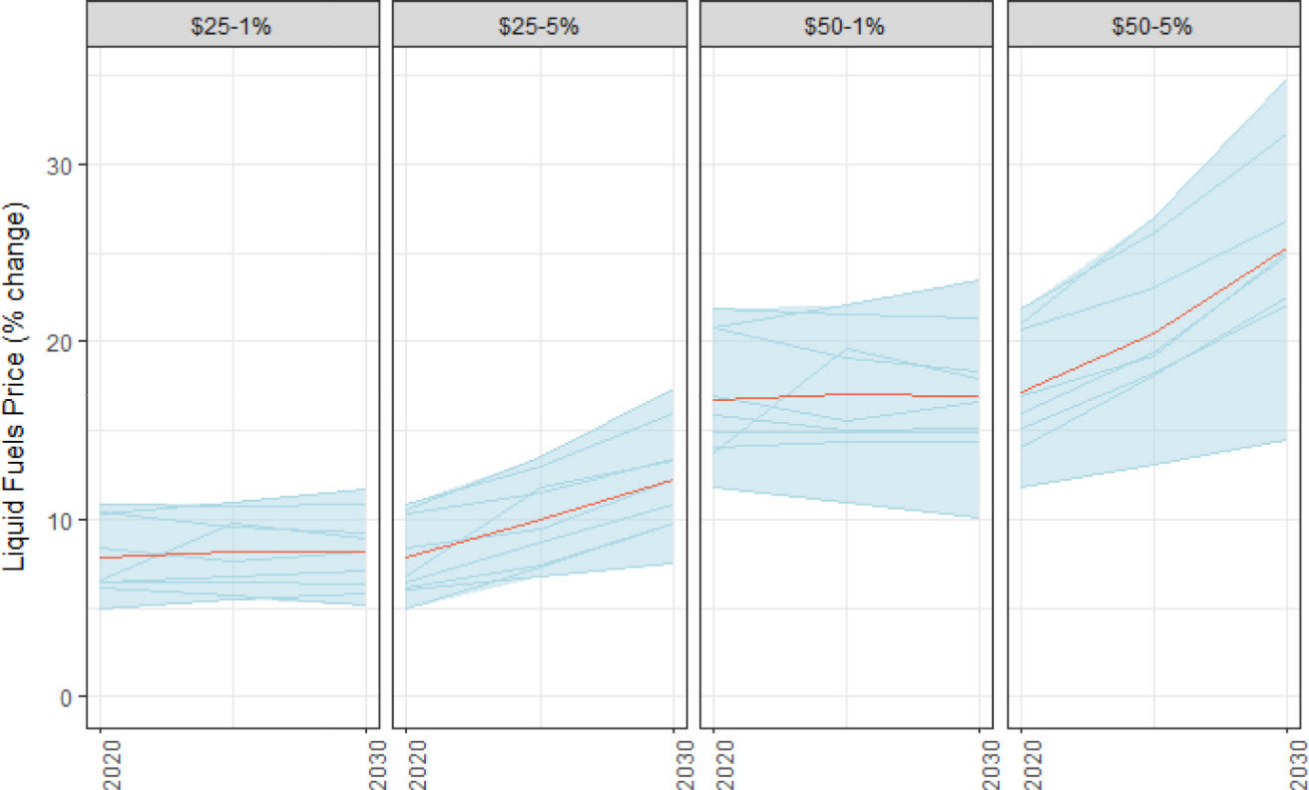
Percent change in liquid fuel (gasoline) prices. Blue bands represent the range of model results, darker blue lines show the individual model results, and the red lines show the average value.

Gasoline prices increase, on average across models, 8% in 2020 under a $25/ton tax and 17% under a $50/ton tax — with relatively tight agreement across models (Fig. 18) (baseline prices are between $2.05 and $3.16/gallon ($2010) in 2020). For taxes with low growth rates, prices impacts from the tax do not rise much further by 2030. These price increases will be felt on top of global oil prices which can (and have been) quite volatile. As noted in Sec. 2, the low variability in price impact is, in part, a reflection of the limited representation of emerging alternatives to gasoline such as electric vehicles in most models.

## 8. Trade and Competitiveness

### 8.1. Trade effects

A central concern about policies aiming to reduce CO_2_ emissions are their effects on trade and competitiveness. As discussed in the previous section, the adoption of a carbon tax raises energy prices. Higher energy prices in turn increase the costs of producing goods. The application of a broad-based carbon tax has implications for U.S. exports and imports, particularly for energy-intensive, trade-exposed (EITE) industries. In the interests of simplicity and transparency, this study applies a carbon tax to only the United States. Such a scenario puts U.S. products at a comparative disadvantage relative to other regions that have not adopted carbon pricing policies. In reality, many other regions of the world, including our main trade partners Canada and Mexico, and some areas of the United States have adopted or are considering price-based measures to reduce GHG emissions (see World Bank and Ecofys 2017). As such, the results here are far closer to a “worst-case” scenario than a likely outcome. Refer to Macaluso *et al*. (this issue) for a more in-depth discussion of trade and leakage effects.

**Figure 19.**
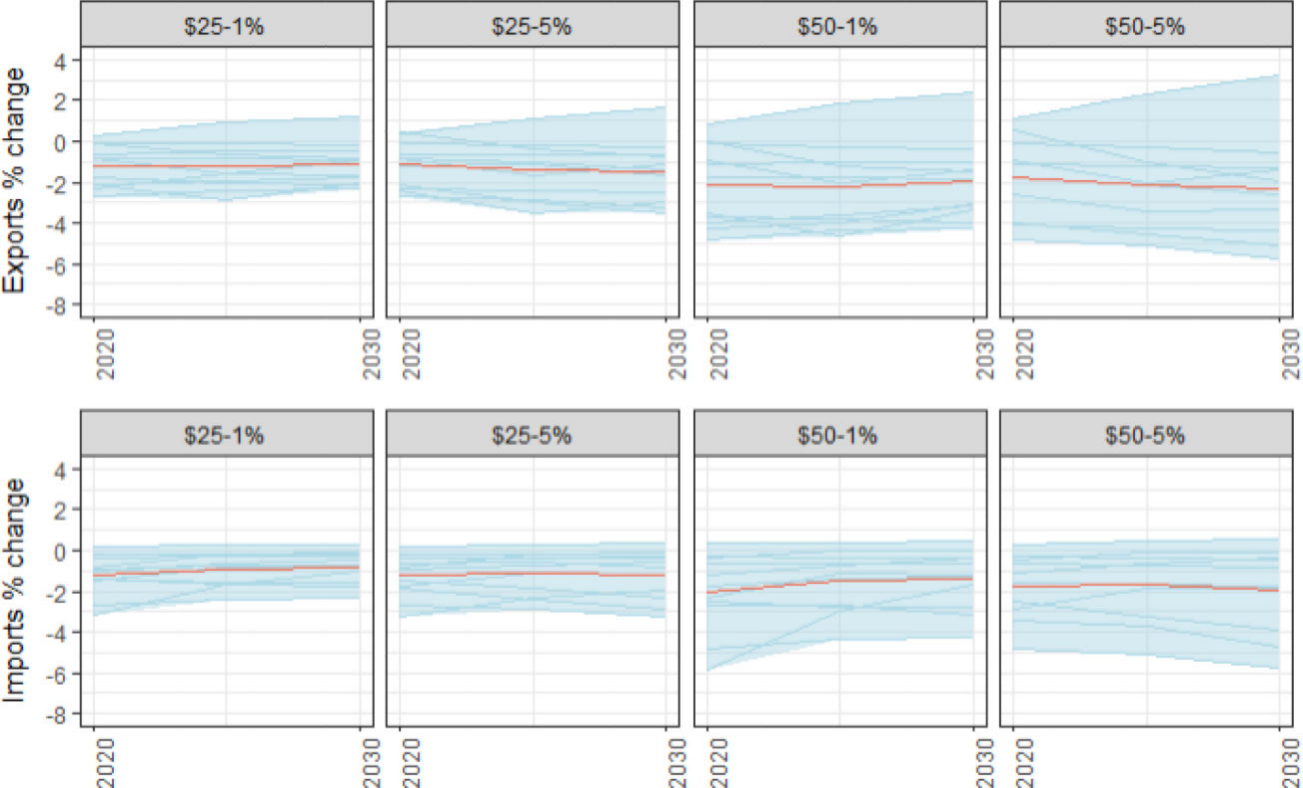
Percent change in exports (top panels) and imports (bottom panels) across all industries.

The change in U.S. exports and imports for all industries is shown in Fig. 19. The multi-model average reduction in U.S. exports across the tax trajectories ranges from 1% to 2.5%. Imports fall by a slightly smaller amount (1–2%). Although the results across the models may differ by several percentage points, the negative effect on imports and exports is robust with only one model showing an increase. The multi-model average remains fairly flat with the exception of a slight decline in exports over time in the $50 at 5% scenario.

**Figure 20.**
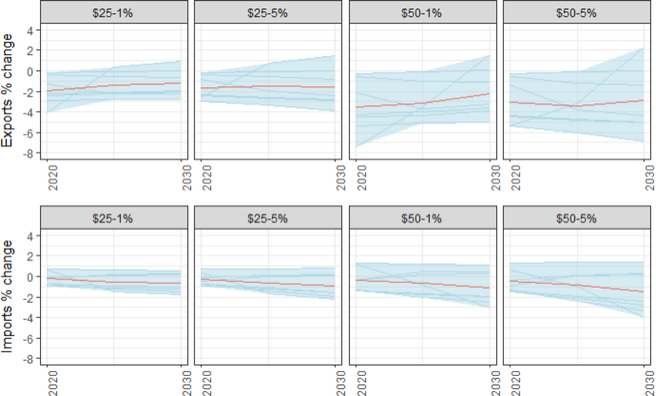
Percent change in exports (top panels) and imports (bottom panels) for EITE industries.

EITE industries are likely to experience greater reductions in exports and lesser reductions in imports than the overall industry average under unilateral carbon tax policies. These industries, which include iron and steel, papermaking, aluminum, glass, and cement, are characterized by a high proportion of energy consumption and trade exposure. Across the models in this study, EITE output represents 5–20% of total industrial output. EITE industries also account for a disproportionate share of industrial emissions, a proxy for their energy intensity. EITE emissions represent 54% of total industrial emissions on average. Trade exposure or intensity, defined as the ratio exports plus imports to the total market size, ranges from 15% to 45% across the models. Figure 20 shows the percentage change in exports and imports for EITE industries. The multi-model average reduction in U.S. EITE exports across the tax trajectories ranges from 1.3% to 3.5%. Imports fall by less than half as much (0.5–1.7%). As would be expected, the EITE sectors face a greater fall in exports than the industry average. Furthermore, U.S. imports of EITE goods fall less as U.S. production is displaced.

### 8.2. Emissions leakage

Under a unilateral carbon tax scenario, some U.S. industries can be placed at a competitive disadvantage relative to regions without emission taxes. Emissions leakage refers to the shift in emissions to regions with less stringent emission policies as production in those regions becomes more cost competitive. Leakage undermines the objective of carbon policies by shifting emissions from one region to another. The leakage rates, defined as the increase in emissions in the rest of the world divided by the emission reduction in the United States, for the two models reporting results in this study are 8% and 19%. This is broadly consistent another multi-model study that estimated leakage values of 5–19% with a mean of 12% (Böhringer *et al*., 2012).

A policy response to address leakage and trade effects is the adoption of border carbon adjustments (BCAs) as a complement to domestic carbon policies. A BCA policy taxes the embodied carbon emissions of imports and rebates the embodied carbon emissions of exports to and from unregulated regions. The aim is to offset the price differential due to the carbon tax between the regulated and unregulated regions. A previous multi-model study showed that BCAs reduce the mean EITE leakage rates by about a third from 12% to 8% (Böhringer *et al*., 2012). McKibbin *et al*. (2018) examine BCAs in the context of revenue recycling schemes and find that revenue recycling plays a critical role in maintaining EITE export strength.

## 9. Other Considerations

Section 2 (above) discusses some of the broad challenges associated with modeling carbon pricing policies. A few other issues are worth noting. First, there is no one model that can address all possible policies or even all possible impacts of a particular policy (SAB, 2017). The models used in this study have been optimized for a range of purposes. Some focus on the electricity sector (REEDS-USREP), whereas others add detail in distributional (DIEM) or intergenerational (CEPE) effects. None of the models captures the benefits of reducing either CO_2_ or co-pollutants. Policymakers should enquire about the strengths and weaknesses of a model when interpreting results and avoid comparing the costs from one model to the benefits from another.

Second, this modeling effort focussed on CO_2_ emissions from fossil fuels in the United States, but climate impacts depend on total GHG emissions globally. A policy could also cover other GHGs (e.g., nitrous oxide, methane, sulfur hexafluoride, hydroflourocarbons). The high global warming potential of some of these gases would lead to much higher equivalent per-ton taxes, but on smaller volumes and with smaller overall economic impacts. The broader coverage of the tax, the more likely it is to drive the most cost-effective reductions and deter behaviors that simply shift from one kind of emissions to another (although some sources present administrative challenges). If hydroflourocarbons are already covered under a Montreal Protocol-type approach, they would not need to be included in a carbon pricing policy. Conversely, a carbon price that increases natural gas usage could increase fugitive methane emissions, undercutting the benefits without other policies in place to prevent such increases. Likewise, a policy that induces greater abatement abroad could have outsize benefits not well reflected in purely domestic emissions levels.

Third, models vary in their representations of technology. As noted earlier, some models like GH-E3 and IGEM do not include CCS technologies, which can be important at higher carbon prices and in scenarios requiring deeper reductions (although costs are very uncertain). Models also vary in their representation of other technological detail as well. Some models such as REEDS-USREP distinguish photovoltaic and concentrating solar power, whereas others represent solar as a single technology (e.g., DIEM). Details like these can impact regional deployment of technology, relative costs, and other policy impacts.

Fourth, carbon taxes address the social costs associated with damages from climate change, but they do not fix other market failures. Carbon prices could be supplemented with investments in R&D, tax credits to spur earlier deployment of technologies such as CCS, policies to increase information available to consumers, and policies to help deploy already cost-effective energy efficiency measures — none of which have been modeled here. In particular, the models generally require that the user prescribes the availability and cost of cost-effective energy efficiency — a highly uncertain parameter but one that is critical to the overall cost and impacts. It is also possible for a carbon tax provision to include tax credits for emissions reductions activities outside covered emissions (the equivalent to offsets in a cap and trade system). Supplemental policies that constrain pathways to compliance, such as renewable energy standards, can increase the overall cost without adding environmental benefits (Burtraw and Palmer, 2013); some of these policies may address other policy goals.

Finally, government spending was held to baseline levels in all of the modeling presented here. Shifts in the net level of government spending as a result of a carbon tax would alter the overall economic impacts.

## 10. Conclusions and Recommendations

Modeling the impacts of a carbon tax is a complex undertaking. The model-to-model variation presented here can help identify results that are robust across models. The range of results is a measure of one type of uncertainty — although uncertainties about technology costs, economic growth, and other factors can be expected to further increase the range of uncertainty.

This uncertainty, however, does not mean that we cannot draw policy insights from these results. Consistent with earlier studies (Fawcett *et al*., 2014), the models suggest that a carbon price can lead to significant reductions in GHG emissions. Higher carbon prices, not surprisingly, lead to greater emissions reductions (especially from the electricity sector), and policymakers can use both the starting point and the escalation rate to target a given goal. While these models produce output for several decades, policymakers would be wise to focus on the results in the next decade or so and give significantly less (or zero) weight to longer term projections of cost, carbon price, or other key metrics. These uncertainties in the long run are not a reason to delay enacting a carbon price in the near future; any pricing policy can be modified as longer-term outcomes evolve, the literature makes clear that delaying action increases the cost to achieve a given level of reductions (McKibbin *et al*., 2014). As we discuss in Sec. 4, these and other models can still be useful to explore uncertainties and evaluate technology pathways in the longer term in ways that can inform investments in research and technology.

Critically, emissions outcomes do not depend upon the use of the revenue to reduce existing taxes; similar reductions will be achieved under very different revenue-recycling scenarios. This is good news, as it gives policymakers freedom to address other policy concerns (such as impacts on low-income households, the federal deficit, and other distortionary taxes) without sacrificing environmental benefits. Economically speaking, a carbon tax is actually two policies that operate in tandem — a price on carbon that reduces emissions and the disposition of revenue associated with that price.

Using revenue to reduce distortionary labor and capital taxes reduces the overall costs relative to lump-sum rebates to households across all models, but the scale of the cost reduction varies significantly across models. While more efficient from the perspective of the larger economy, these revenue recycling methods may raise equity concerns for policymakers as they have regressive impacts in most models — impacting the lowest income quintiles most (Fig. 8). However, the use of revenue is not an all or nothing approach, and hybrid methods may be able to help balance efficiency and equity (as well as other considerations such as salience and simplicity).

As has been seen in many earlier modeling efforts, the impacts of a carbon tax will vary greatly by sector. There was broad agreement in the models that the majority of the emissions reductions in the short term will come from the electricity sector. However, we do not have a clear understanding of the degree to which a carbon price can drive reductions in other sectors in the mid to long term.

Under the technology assumptions used in this model, carbon prices at $25/ton or even lower cause significant shifts away from coal as an energy source. Low cost zero-carbon energy (i.e., wind under the assumptions in the models used here) increases, sometimes drastically, as the carbon price increases but other nonfossil energy sources (biomass and solar) are likely to increase or decrease depending upon relative costs of competing forms of energy (wind and natural gas). Policymakers should be aware of this sensitivity and request a range of technology and fuel costs be analyzed whenever feasible.

Increased prices for energy from fossil fuels are an inescapable component of a carbon tax. The models analyzed here, absent other policy measures, show increases in the price of electricity, home heating fossil fuels, and gasoline. As with emissions, increased stringency of the tax leads to greater price increases. These cost increases for consumers can be offset with recycling of the revenue as discussed above or by adopting complementary policies to increase deployment of energy efficiency measures and provide transportation and heating alternatives.

McFarland *et al*. (this issue) discuss possible directions for future research modeling carbon taxes. Here we focus on a number of technical recommendations relevant to policymakers working with these models in the future.

### Carbon taxes should be analyzed with the most current economic and technological information feasible

As noted earlier, these costs and impacts of a carbon tax policy are sensitive to key parameters such as economic growth, natural gas prices, energy efficiency costs, and zero carbon energy costs. Some of these costs change rapidly on a year-to-year basis. Modeling of a carbon tax with the HAIKU model in 2012 suggested that coal generation would be replaced by gas and renewables at a carbon price of roughly $50/ ton (rising at 5%/year). With more recent costs, this transition now occurs in HAIKU for a price trajectory starting at $25/ton (Palmer *et al*., 2018). Even in the development of this study, many modelers switched from calibration with AEO2015 to AEO2016 or related updated technology costs. This led to significant shifts in the model output — reflecting several large changes in the AEO. This sensitivity highlights the uncertainties in the projections of these models but also suggests that policymakers should ensure that modeling efforts do their best to capture the most recent information on key parameters. For example, if prices are falling rapidly for solar photovoltaics or batteries, it will be important to include the most current information in the model.

### Policymakers should request multiple sensitivities, ideally from multiple models

Even with the most current information, uncertainties will remain about economic growth, fossil fuel prices, technology costs, and other critical parameters. While models are generally not able to explore the full range of uncertainty (especially in the short time windows under which policies are generally formed), a robust set of sensitivities can provide policymakers a better sense of the range of possible outcomes for any given tax level and design. Policymakers can then discuss the potential likelihood of any given sensitivity case with technical experts (again, keeping in mind that our ability to predict such things is not strong). Similarly, the results from this study, and the long history of the EMF, show that two models can produce qualitatively different results from the same starting assumptions. Examination of multiple models provides a more robust picture from which to generate policy design insights. In contexts in which time and/or resources prevent the use of a suite of models, the results from this and other EMF studies can be used to help place the results from a single model or smaller suite of models in context. However, even an analysis with multiple models does not really provide a complete accounting of the full range of outcomes. Technological and economic shocks over time, unanticipated by modelers today, increase the range of possible outcomes in terms of both emissions levels and costs.

### Models are best for insight, not foresight

There is a very natural temptation to want to use these models to form expectations about costs and impacts of carbon price policies. However, because of the inherent challenges and uncertainties, policymakers are on steadier ground if they ask first what modeling can teach us about tradeoffs between policy choices, key sensitivities, and other key design decisions and then design the policy to be robust to variations in both assumptions and outcomes.

### Request estimates of benefits

Prior work and the modeling here demonstrate that the benefits from reducing conventional air pollution and climate impacts are significant. Only one model in this study directly reported economy-wide changes in key air pollutants. Modelers could develop capacity to produce this output, and tools exist to calculate emissions impacts after the fact (Abt, 2017).

### There will be surprises

Oscar Wilde once observed: “To expect the unexpected shows a thoroughly modern intellect” (Wilde, 1899). Climate policy is no different than other kinds of complex policies where the implemented policy is likely to work out differently than originally anticipated. The long-time scale and complexity of the sectors involved only enhances the need to think about policy designs that balance certainty for planning purposes with the need for mechanisms (either in the executive branch or through future legislative action) to make adjustments over time (Burtraw and Palmer, 2013; Aldy, 2017; Hafstead *et al*., 2017; Murray *et al*., 2017).

### Modeling can be improved with investment

Given the scale and importance of climate policy, investments in models and supporting data are an important way to improve decisions about policy design. Like most efforts of this type, sustained support for institutional capacity in federal agencies and for other researchers is the best way to continued improvements in the tools available. In particular, we recommend more investment in understanding the costs of reducing emissions outside the power sector and for energy efficiency measures (Barron, 2018). As more carbon pricing policies are deployed globally, there will also be more opportunities to understand how modeled responses may differ from those observed in the real world.

### Invest in policy staff that can engage with the technical details of carbon policy analysis

Training in economic modeling is scarce among legislative staff (although better represented among federal agency staff). For decision makers to make the best use of modeling, there is no substitute for staff that understand how these models work and what they can and cannot do. Outside experts from academia, think tanks, and industry play an important role, but staff who understand both the technical material and the decision-makers’ priorities can translate and interpret the information.

## Supplementary Material

supplemental

## Figures and Tables

**Table 1.** Cumulative emissions reductions by price path (in GTCO_2_ and percentage) 2020–2030.

Scenario	Gigatons of CO_2_ emissions reduced			Percentage decrease from reference		
		
	Minimum	Average	Maximum	Minimum (%)	Average (%)	Maximum (%)
$25–1%	6.3	10.5	17.6	11	18	30
$25–5%	7.6	12.3	20.4	13	21	35
$50–1%	11.2	17.2	28.3	19	29	48
$50–5%*	13.0	17.9	22.2	22	30	38

*Note*:

* As in Fig. 1, the model with the most aggressive reductions did not report results for the $50–5% scenario, thus the reported maximum reductions in the $50–1% scenario are greater than those in the $50–5% scenario.

**Table 2.** Cumulative gross carbon tax revenue from 2021 to 2030 under lump-sum recycling (trillion $2010).

Scenario	Minimum	Average	Maximum
$25–1%	0.97	1.18	1.25
$25–5%	1.11	1.41	1.53
$50–1%	1.40	2.03	2.27
$50–5%	2.19	2.48	2.72

## Footnotes

1 All monetary amounts are $2010 unless otherwise noted.

2 At press, this policy is being re-proposed.

3 See Chen *et al*. (this issue) for an analysis of the impact of alternative reference case scenarios on emissions outcomes using the E3 model.

4 For the models in this paper, six had perfect foresight, three were recursive dynamic (optimizing only over the short term), and two models had a mix (see Babiker *et al*. (2009) for a discussion of tradeoffs). Recursive dynamic models still have an aspect of unrealistic foresight as they assume that current conditions will continue into the future.

5 Early research on directed technological change and climate policy was theoretical and “not designed for quantitative analysis.” More recent work has shown, in a three-sector model, that including induced innovation significantly “increases the effectiveness of the [carbon tax] policy at reducing emissions, resulting in a 19.2 percent decrease in the size of the carbon tax required to reduce emissions by 30 percent in 20 years” (Fried, 2018).

6 EPA’s Greenhouse Gas Inventory currently lists 2005 fossil fuel emissions as 5746.9 MMT CO_2_.

7 These estimates are approximate as model calibrations mean that emissions may differ slightly in 2005, affecting the effective emissions reduction.

8 Three models showed a small amount of anticipatory reductions before 2020, not reported here.

9 Since not all of the models participating in EMF 32 include non-CO_2_ emissions or land and forest carbon included in the NDC target, in this study we calculated the gross fossil energy CO_2_ emissions target that are consistent with the net GHG emissions target assuming a steady U.S. land use, land-use change, and forestry sink through 2025, and equal percentage reductions in CO_2_ and non-CO_2_ emissions. Given these assumptions, a target of 26% below 2005 levels for U.S. net GHG emissions can be achieved with fossil energy CO_2_ emissions that are 23% below 2005 levels.

10 Prices required to meet annual targets, such as the 26% reduction in 2025 target, depend importantly on the year the carbon tax begins. As shown in Chen and Hafstead (2016), both the price needed and the cost of the policy increase with each year of delay due to higher capital stocks in polluting industries and the presence of capital adjustment costs.

11 For more detailed results from the electricity sector, see Bistline *et al*. (2018).

12 A number of countries have recently set goals for 100% of new vehicles to be electric (Petroff, 2017).

13 We only show revenues under household rebates because the size of revenues is similar across recycling methods.

14 Note that the model with the lowest emissions and carbon tax revenue in the $50 at 1% scenario (G-cubed) did not run the $50 at 5% scenario. Also note that declines in gross carbon revenues are only possible if emissions fall more quickly than the rate of price increase; most models do not predict this. For more detail, see McFarland *et al*. (this issue).

15 To convert from 2010 dollars to 2017 dollars with a GDP deflator, just multiply the values in the figures and Table 2 by 1.12 (U.S. Bureau of Economic Analysis, 2017).

16 Economists have debated the possibility of a double dividend for nearly 30 years (see Goulder (1995) for an early discussion). For obvious reasons, the idea that environmental benefits can be achieved with no overall macroeconomic cost is appealing to many. We emphasize, however, that the existence of a double dividend is as much a statement about the inefficiencies of the tax system as it is about the direct or indirect costs of carbon taxes.

17 McFarland *et al*. (2018) report the same results over the 2015–2040 time frame.

18 In principle, welfare could also include environmental benefits (Paltsev and Capros, 2013), but those are not modeled explicitly in this study.

19 The U.S. Interagency Working Group (IWG) on the Social Cost of Greenhouse Gases estimate the social cost of carbon dioxide (SC-CO_2_) in 2025 using a 3% discount rate to be $48 ($2010, original report in $2007) dollars per metric ton of CO_2_ (IWG, 2016). The IWG also estimated the SC-CO_2_ in 2025 using discount rates of 5% ($15) and 2.5% ($71). Valuing the 2025 emissions reductions under the $25–5% scenario using these alternate values of the SC-CO_2_ suggests lower (and possibly negative) or higher net benefits, respectively. A large and growing literature estimates the effects of climate change; but estimates vary widely and debate surrounds how they should be applied for policymaking. A full treatment of this issue is beyond the scope of this paper, but a few points are worth noting here. For example, the global SCC of $48 per ton applied above captures only some of the damages associated with climate change (Howard, 2014; National Academies of Sciences, Engineering, and Medicine, 2017), and it does not account for willingness to pay to avoid risks associated with bad or catastrophic climate outcomes. Accounting for these factors could significantly increase SCC estimates. Other recent estimates of the global benefits of CO_2_ reductions are often over $100/ton (van den Bergh and Botzen, 2014; Moore and Diaz, 2015).

20 E.O. 13783, issued in March of 2017 (White House, 2017), disbanded the IWG, withdrew the technical support documents (TSDs) describing the global social cost of GHGs as “no longer representative of government policy,” and directed that any future use of the social cost of carbon to monetize the value of changes in greenhouse gas emissions resulting from regulations must be consistent with the guidance contained in OMB Circular A-4 (OMB, 2003). EPA’s “*Regulatory Impact Analysis for the Review of the Clean Power Plan: Proposal*” released in October 2017, includes updated interim SC-CO_2_ values (EPA, 2017b). Citing Circular A-4’s guidance that analysis of regulations “should focus on benefits and costs that accrue to citizens and residents of the United States,” these interim values include a domestic only SC-CO_2_ estimates, and in accordance with Circular A-4’s guidance that when an agency “evaluate[s] a regulation that is likely to have effects beyond the borders of the United States, these effects should be reported separately,” EPA separately reported global SC-CO_2_ estimates. EPA notes that these interim values “will be used in regulatory analysis until improved domestic estimates can be developed, which would take into consideration the recent recommendations from the National Academies of Sciences, Engineering, and Medicine for a comprehensive update to the current methodology to ensure that the SC-CO_2_ estimates reflect the best available science” (National Academies of Sciences, Engineering, and Medicine, 2017). The average domestic SC-CO_2_ contained in EPA’s “*Regulatory Impact Analysis for the Review of the Clean Power Plan: Proposal*,” for emissions occurring over 2020–2030 ranges from $9 to $10 per metric ton of CO_2_ using a 2.5% discount rate, from $6 to $7 per ton using a 3% discount rate, and is around $1 per ton of CO_2_ using a 7% discount rate ($2010). The average global SC-CO_2_ contained in EPA’s “*Regulatory Impact Analysis for the Review of the Clean Power Plan: Proposal*,” for emissions occurring over 2020–2030 ranges from $65 to $75 per metric ton of CO_2_ using a 2.5% discount rate, from $43 to $52 per ton using a 3% discount rate, and from $5 to $7 per ton of CO_2_ using a 7% discount rate ($2010). Using any of these alternate values of the SC-CO_2_ can suggest either lower (and possibly negative) or higher net benefits.

21 “Estimation of the net damages per ton of CO_2_ emissions to the United States alone, beyond the approximations done by the IWG, is feasible in principle; however, it is limited in practice by the existing SC-IAM methodologies, which focus primarily on global estimates and do not model all relevant interactions among regions. It is important to consider what constitutes a domestic impact in the case of a global pollutant that could have international implications that impact the United States. More thoroughly estimating a domestic SC-CO_2_ would therefore need to consider the potential implications of climate impacts on, and actions by, other countries, which also have impacts on the United States” (National Academies of Sciences, Engineering, and Medicine, 2017).

22 Kotchen (2016) shows that the efficient Pareto optimal outcome is for each country to internalize the global SCC, while the use of a domestic SCC is consistent with a Nash equilibrium in a static one-shot game. However, taking into account the real-world institutional context in which climate policy and international negotiations take place, by either assuming countries interact repeatedly, and are concerned about the future (i.e. moving from a static to a repeated game); or, by taking into account the international relations argument that through leadership and leverage countries will respond to each other’s choice of emissions (i.e. using a conjectural variations approach), the paper shows that internalizing the global SCC can be in a country’s self-interest, and a country’s preferred SCC will be greater than the domestic SCC, though not necessarily equal to the global SCC.

23 As discussed in Goulder and Hafstead (2018), the effect of changes in the price of consumption goods is termed the “use-side” effect. The effect of changes in income is termed the “source-side” effect. In general, revenue recycling affects the “source-side” through additional transfer income (e.g., lump-sum rebates) or labor or capital income (e.g., reductions in labor or capital tax rates). However, revenue recycling options may also affect the change in prices. Specifically, capital tax reductions spur investment which dampens the effect of carbon taxes on the price of consumption goods

24 Low-income households without labor or capital income have smaller welfare changes under capital tax cuts due to lower source-side impacts as discussed in the previous footnote.
