# Supplementary material for: POLICY INSIGHTS FROM THE EMF 32 STUDY ON U.S. CARBON TAX SCENARIOS
Source: Clim Chang Econ (Singap). Author manuscript; Available in PMC 2019 Aug 19. (PMC6699640; doi:10.1142/S2010007818400031)
Supplement: supplemental [file NIHMS963076-supplement-supplemental.pdf]

January 25, 2018

Supplementary Material

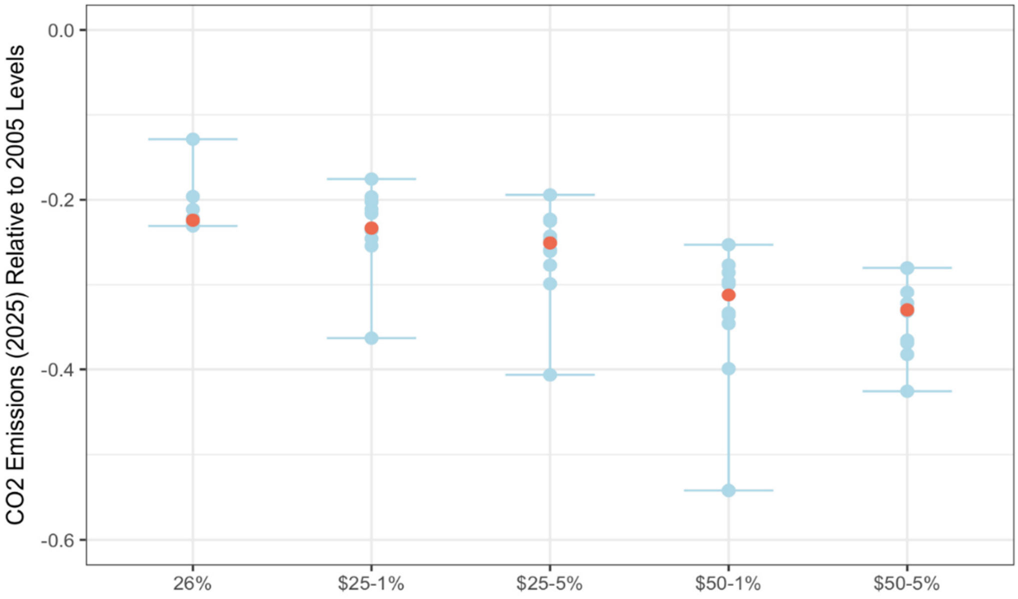

Figure S1. Change in 2025 Fossil Fuel CO2 Emissions from 2005 levels. Blue whiskers show the range and the dots lines show the average.

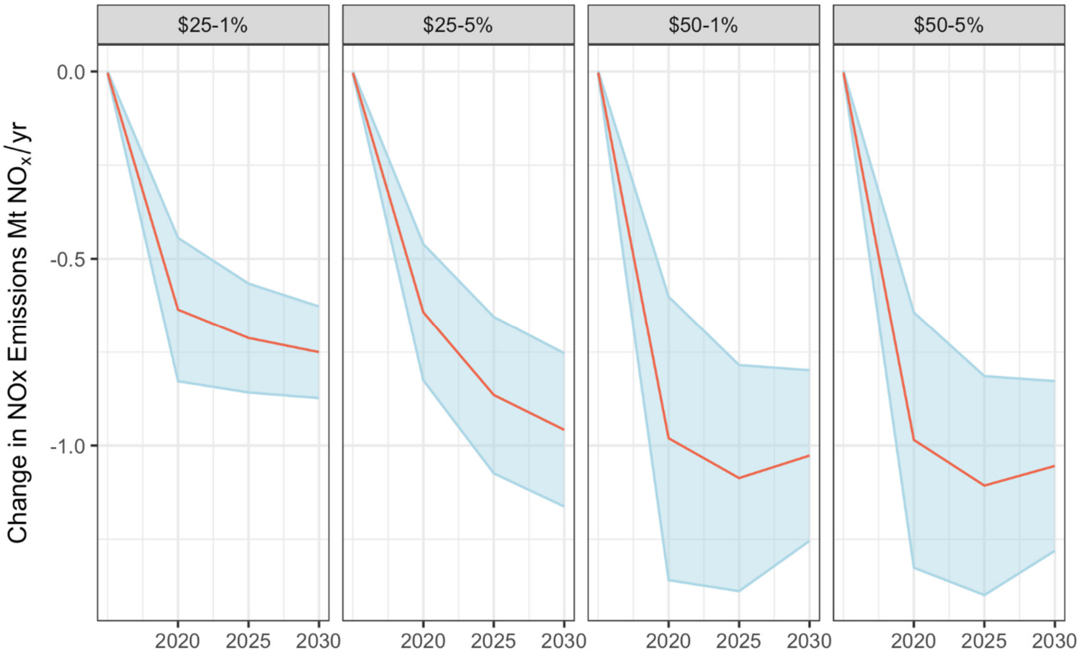

Figure S2. Nitrogen dioxide reductions from the electricity sector relative to business as usual (Mt NOx/yr) by year under four carbon tax stringencies. Blue bands represent the range of model results, points show the individual model results and the red lines show the median value. Only 2 models reported this variable. This figure represents reductions beyond the 16-20% decline from current levels in the reference case by 2030.

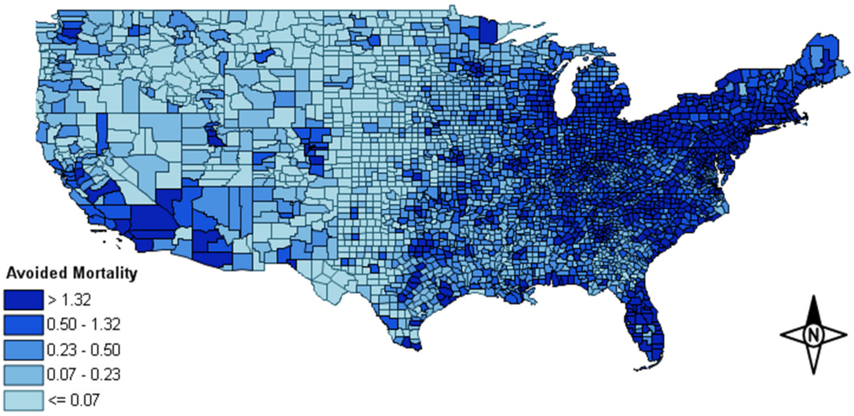

Figure S3: Illustrative distribution of electricity sector-derived air quality co-benefits in 2025 from a $25 carbon tax rising at 5%/year. Avoided premature mortality health benefits estimated using Krewski et al. (2009) and the BENMAP model (via the COBRA model) from median SO_2 and NO_x reductions in the electricity sector (other air quality improvements from other pollutants and other sectors not reflected).

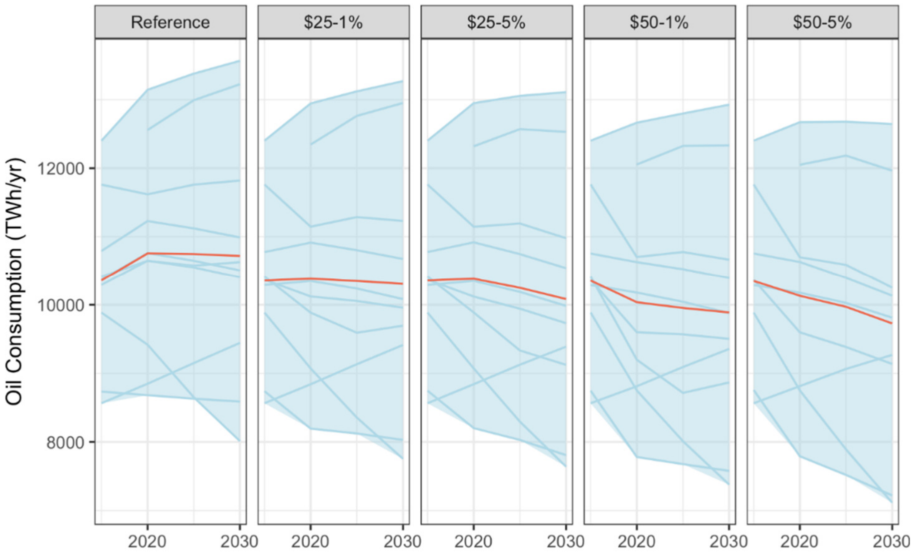

Figure S4. Oil consumption (TWh/yr) under four carbon tax stringencies.

Supplementary Information on COBRA Analysis

Health benefits were analyzed using the Co-Benefits Risk Assessment COBRA model (v 3.0) on 2025 base year data. Reductions were applied to Fuel Combustion Electric Utilities|Coal on a tonnage basis to SO_2 and NOx based on mean decreases from the models reporting this variable for the $25 tax rising at 5%/year. All other parameters were maintained at model defaults.
